# Hyperpolarized relaxometry based nuclear *T*_1_ noise spectroscopy in diamond

**DOI:** 10.1038/s41467-019-13042-3

**Published:** 2019-11-14

**Authors:** A. Ajoy, B. Safvati, R. Nazaryan, J. T. Oon, B. Han, P. Raghavan, R. Nirodi, A. Aguilar, K. Liu, X. Cai, X. Lv, E. Druga, C. Ramanathan, J. A. Reimer, C. A. Meriles, D. Suter, A. Pines

**Affiliations:** 1 Department of Chemistry, and Materials Science Division Lawrence Berkeley, National Laboratory University of California, Berkeley, CA 94720 USA; 2 Department of Physics and Astronomy, Dartmouth College, Hanover, NH 03755 USA; 3 Department of Chemical and Biomolecular Engineering, and Materials Science Division Lawrence, Berkeley National Laboratory University of California, Berkeley, CA 94720 USA; 4 Department of Physics and CUNY-Graduate Center, CUNY-City College of New York, New York, NY 10031 USA; 5 Fakultät Physik, Technische Universität Dortmund, D-44221 Dortmund, Germany

**Keywords:** Quantum information, Quantum metrology, NMR spectroscopy

## Abstract

The origins of spin lifetimes in quantum systems is a matter of importance in several areas of quantum information. Spectrally mapping spin relaxation processes provides insight into their origin and motivates methods to mitigate them. In this paper, we map nuclear relaxation in a prototypical system of ${}^{13}{\rm{C}}$ nuclei in diamond coupled to Nitrogen Vacancy (NV) centers over a wide field range (1 mT-7 T). Nuclear hyperpolarization through optically pumped NV electrons allows signal measurement savings exceeding million-fold over conventional methods. Through a systematic study with varying substitutional electron (P1 center) and ${}^{13}{\rm{C}}$ concentrations, we identify the operational relaxation channels for the nuclei at different fields as well as the dominant role played by ${}^{13}{\rm{C}}$ coupling to the interacting P1 electronic spin bath. These results motivate quantum control techniques for dissipation engineering to boost spin lifetimes in diamond, with applications including engineered quantum memories and hyperpolarized ${}^{13}{\rm{C}}$ imaging.

## Abstract (web summary)

Nuclear spins in diamond have applications in quantum technologies and NMR methods but their performance can be limited by relaxation processes that are difficult to characterise. Ajoy et al. develop a *T*_1_ noise spectroscopy method to identify the dominant relaxation channel and propose a mitigation strategy.

## Introduction

The power of quantum technologies, especially those for information processing and metrology, relies critically on the ability to preserve the fragile quantum states that are harnessed in these applications^1^. Indeed noise serves as an encumbrance to practical implementations, causing both decoherence as well as dissipation of the quantum states^2,3^. Precise spectral characterization of the noise opens the door to strategies by which it can be effectively suppressed^4,5^—case in point being the emergence of dynamical decoupling techniques that preserve quantum coherence by periodic driving^6^. In these cases, quantum control sets up a filter that decouples components of noise except those resonant with the exact filter period^7^, allowing spectral decomposition of the dephasing noise afflicting the system. Experimentally implemented in ion traps^8^, superconducting qubits^9^, and solid-state NMR^10^, this has spurred development of Floquet engineering to enhance ${T}_{2}$ decoherence times by over an order of magnitude in these physical quantum device manifestations^11–13^.

Methods that analogously spectrally fingerprint ${T}_{1}$ relaxation processes, on the other hand, are more challenging to implement experimentally. If possible, however, they could reveal the origins of relaxation channels, and foster means to suppress them. Applications to real-world quantum platforms are pressing: relaxation in Josephson junctions and ion-trap qubits, for instance, occur due to often incompletely understood interactions with surface paramagnetic spins^14^. Relaxation studies are also important in the context of coupled quantum systems, such as those built out of electronic and nuclear spins. In the case of diamond Nitrogen Vacancy (NV) center electronic qubits coupled to ${}^{13}{\rm{C}}$ nuclei^15^, for instance, a detailed understanding of nuclear relaxation can have important implications for quantum sensing^16^: engineered NV-${}^{13}{\rm{C}}$ clusters form building blocks of quantum networks^17^, are the basis for spin gyroscopes^18^, and are harnessed as quantum memories in high-resolution nano-MRI probes^19^. Nuclear ${T}_{1}$ lifetimes are not dominated by phonon interactions, but instead are set by couplings with the intrinsic electronic spin baths themselves—a complex dynamics that is often difficult to probe experimentally. Indeed only a small proportion of ${}^{13}{\rm{C}}$ spins can be addressed or readout via the NV centers, as also the direct inductive readout of these spins suffer from extremely weak signals. Moreover, as opposed to ${T}_{2}$ noise spectroscopy carried out in the rotating frame^13^, probing of ${T}_{1}$ processes have to be performed in the laboratory frame. This necessitates the ability to probe relaxation behavior while subjecting samples to widely varying magnetic field strengths.

In this paper, we develop a method of hyperpolarized relaxometry that overcomes these instrumentational and technical challenges. We measure ${T}_{1}$ relaxation rates of ${}^{13}{\rm{C}}$ spins in diamond samples relevant for quantum sensing with a high density of NV centers. Our ${T}_{1}$ noise spectroscopy proceeds with high resolution and over four decades of noise spectral frequency, revealing the physical origins of the relaxation processes. While experiments herein are demonstrated for ${}^{13}{\rm{C}}$ spins in electron-rich diamond, these results are potentially more widely reflective of relaxation processes operational in other systems, including Si:P^20^, wide bandgap materials such as SiC^21,22^, and diamond-based quantum simulator platforms constructed out of 2D materials such as graphene and hBN^23–25^. These results are also pertinent for producing and maintaining polarization in hyperpolarized solids, for applications employing hyperpolarized nanoparticles of Si or diamond as MRI tracers^26,27^, and in the relayed optical DNP of liquids mediated through nanodiamonds^28^, since in these applications ${T}_{1}$ relaxation bounds the achievable polarization levels.

## Results

### ${}^{13}{\rm{C}}$ Hyperpolarized relaxometry

**Fig. 1:**
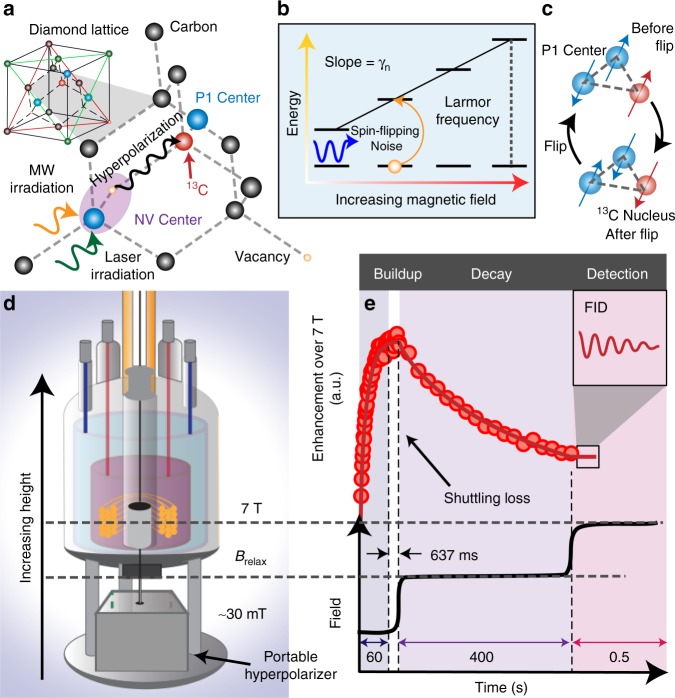
Principle. **a** System consisting of ${}^{13}{\rm{C}}$ nuclear spins in diamond hyperpolarized via NV centers allowing their direct measurement by bulk NMR. Lattice also contains electronic spin bath of P1 centers. **b** Changing magnetic field allows probing of spin-flipping noise that is resonant with the carbon Larmor frequency. **c** Dominant ${T}_{1}$ relaxation mechanism via three-body flip-flops with pairs of P1 center electrons. **d** Experimental platform. Portable hyperpolarizer is installed in a rapid field cycling device capable of sweeping between 10 mT-7 T in the fringe field of a NMR magnet. **e** Time sequence. Lower panel shows the schematic steps of laser-driven optical ${}^{13}{\rm{C}}$ hyperpolarization for $\sim$60 s at ${B}_{{\rm{pol}}}\approx$ 30 mT, rapid shuttling ($<$1 s) to the field of interest ${B}_{{\rm{relax}}}$, relaxation, and subsequent high-field detection at 7 T. Upper panel displays typical data for 200 µm microdiamond powder, where ${B}_{{\rm{relax}}}=$ 2 T. ${}^{13}{\rm{C}}$ NMR signal amplitude (points) is quantified by its enhancement over the 7 T Boltzmann signal. Signal growth and decays are fitted to stretched exponentials (solid lines).

Key to our technique is the hyperpolarization of $${}^{13}{\rm{C}}$$ nuclei at room temperature, allowing the rapid and direct measurement of nuclear spin populations via bulk NMR^28^. Dynamic nuclear polarization (DNP) is carried out by optical pumping and polarizing the NV electrons to large values ($$> $$10%^31,32^) and subsequently transferring this to $${}^{13}{\rm{C}}$$ nuclei (Fig. 1a). This routinely leads to nuclear polarization levels $$\gtrsim$$ 0.5%. In a high-field (7 T) NMR detection spectrometer, for instance, the signals are enhanced by factors exceeding $$\varepsilon \sim$$ 300–800 times the Boltzmann value^28^, boosting measurement times by 10^5^–10^6^, and resulting in high single-shot detection SNRs. This permits $${T}_{1}$$ spectroscopy experiments that would have otherwise been intractable. Hyperpolarization is equally efficiently generated in single crystals as well as randomly oriented diamond powders, and both at natural abundance as well as enriched $${}^{13}{\rm{C}}$$ concentrations. The hyperpolarized samples are interfaced to a home built field cycler instrument^33^ (see Fig. 1d and video in ref. ^34^) that is capable of rapid and high-precision changes in magnetic field over a wide 1 mT–7 T range (extendable in principle from 1 nT–7 T), opening a unique way to peer into the origins of nuclear spin relaxation.

Figure 1d, e schematically describe the experiment. Hyperpolarization in the ${}^{13}{\rm{C}}$ nuclei is affected by optical pumping at low fields, typically ${B}_{{\rm{pol}}} \sim$ 40 mT, followed by rapid transfer to the intermediate field ${B}_{{\rm{relax}}}$ where the spins are allowed to thermalize (see Fig. 1c), and subsequent bulk inductive measurement at 7 T. Experimentally varying ${B}_{{\rm{relax}}}$ allows one to probe field-dependent lifetimes ${T}_{1}({B}_{{\rm{relax}}})$, and through them noise sources perpendicular to ${{\bf{B}}}_{{\rm{relax}}}$ and resonant with the nuclear Larmor frequency ${\gamma }_{n}{B}_{{\rm{relax}}}$ (Fig. 1b). Here, ${\gamma }_{n}=10.7$ MHz T${}^{-1}$ is the ${}^{13}{\rm{C}}$ gyromagnetic ratio. This allows the spectral decomposition of noise processes that spawn ${T}_{1}$ relaxation. For instance pairs of substitutional nitrogen impurities (P1 centers) undergoing flip-flops (Fig. 1c) can apply on the ${}^{13}{\rm{C}}$ nuclei a stochastic spin-flipping field that constitutes a relaxation process.

**Fig. 2:**
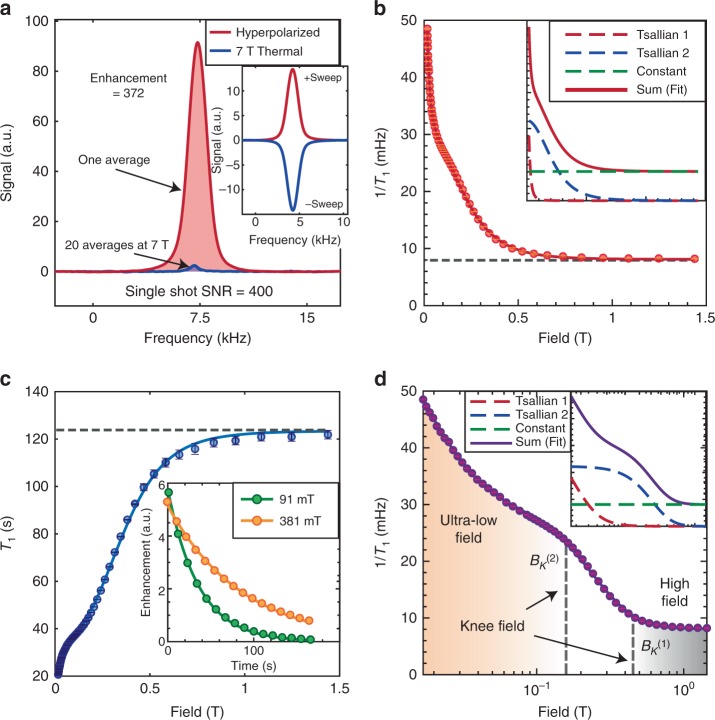
Hyperpolarized relaxometry applied to a 10% ${}^{13}{\rm{C}}$-enriched single crystal (sample 6 in Table 1). **a** Signal gains due to hyperpolarization under optimal conditions at ${B}_{{\rm{pol}}}\approx$ 36 mT. Red line shows a single-shot hyperpolarized signal (SNR $\approx$ 400) after 60 s of optical pumping. Blue line is the 7 T thermal signal after 20 averages, allowing us to quantify signal enhancement from DNP $\approx$372 over 7 T, a time saving by $\approx\!\!1{0}^{6}$ for equal SNR. Inset: Exemplary signals at ${B}_{{\rm{pol}}}\approx$ 36 mT under low-to-high (high-to-low) frequency sweeps leading to positive (negative) ${}^{13}{\rm{C}}$ hyperpolarization. **b** Relaxation rate ${R}_{1}=1/{T}_{1}$ obtained from relaxometry over a wide-field range 20 mT–1.5 T. We observe a rapid growth in relaxation rate below a knee field of 0.5 T, and saturation at higher fields. Inset: Data can be fit to two Tsallian functions, which we ascribe to be originating from inter-${}^{13}{\rm{C}}$ couplings and interactions to the P1 spin bath. **c** Spin lifetimes as a function of field, showing significant boost in nuclear ${T}_{1}$ beyond the knee field, approaching a lifetime $\approx$ 2.1 min. Inset: Typical relaxation data at two representative fields showing monoexponential character. **d** Logarithmic scale data visualization, displaying a more equanimous sampling of experimental points, and the knee fields inflection points ${B}_{K}^{(1,2)}$. Inset: Decomposition into the constituent Tsallians. Error bars in all panels are obtained from monoexponential fits.

Optical excitation for hyperpolarization involves 520-nm irradiation at low power (~80 mW mm^−2^) applied continuously for ~40 s. Microwave (MW) sweeps, simultaneously applied across the NV center ESR spectrum, transfer this polarization to the ${}^{13}{\rm{C}}$ spins (see Fig. 2a)^28,35^. To prevent excessive heating during optical irradiation, samples are immersed under water, with the powdered diamonds precipitated in the NMR tube. The hyperpolarization is carried out at room temperature, and at the relatively low optical power densities we employ, we do not observe sample heating (temperature $<$ $100^{\circ}$ C). DNP occurs in a manner that is completely independent of crystallite orientation. All parts of the underlying NV ESR spectrum produce hyperpolarization, with intensity proportional to the underlying electron density of states. The polarization sign depends solely on the direction of MW sweeps through the NV ESR spectrum (see Fig. 2a inset). Physically, hyperpolarization arises from partly adiabatic traversals of a pair of Landau–Zener (LZ) crossings in the rotating frame that are excited by the swept MWs. For a more detailed exposition of the DNP mechanism, we point the reader to Ref. ^36^.

### Low-field DNP

Low-field hyperpolarization is hence excited independent of the fields $${B}_{{\rm{relax}}}$$ under which relaxation dynamics is to be studied. There is significant acceleration in acquisition time since optical DNP obviates the need to thermalize spins at high fields where $${T}_{1}$$ times can be long (for some samples $$> $$30 min). Gains averaging time are $$\approx {\varepsilon }^{2}\frac{{T}_{1}({\rm{7T}})}{{T}_{1}({B}_{{\rm{pol}}})}$$, which in our experimental conditions exceeds five orders of magnitude. In Fig. 2a for instance on a 10% enriched single crystal, we obtain large DNP enhancements $$\varepsilon =$$ 380, and high single shot SNR $$\approx$$ 400. It also reflects the inherently high DNP efficiency: every NV center has surrounding it $$\sim$$10^5^ nuclear spins, which we polarize to a bulk value (averaged over all $${}^{13}{\rm{C}}$$ nuclei) of 0.37% employing just 3000 MW sweeps, indicating a transfer efficiency of $$\approx$$12.3% per sweep per fully polarized nuclear spin, assuming full NV polarization. Harnessing this large signal gain allows us to perform relaxometry at a density of field points that are about two orders of magnitude greater than previous efforts^37–39^. Such high-resolution spectral mapping (for instance 55 field points in Fig. 2) can transparently reveal the underlying processes driving nuclear relaxation. Indeed, in future work, use of small flip-angle pulses might allow one to obtain the entire relaxation curve with a single round of DNP, and thus the ultrafast relaxometry of the nuclei.

Our experiments are also aided by technological attributes of the DNP mechanism. DNP is carried out under low fields and laser and MW powers, and allows construction of a compact hyperpolarizer device that can accessorize a field cycling instrument^40^ (see ^41^ for video of hyperpolarizer operation). The wide range (1 mT–7 T) field cycler is constructed over the 7 T detection magnet, and affects rapid magnetic field changes by physically transporting the sample in the axial fringe-field environment of the magnet^33^. This is accomplished by a fast (2 m s^−1^) conveyor belt actuator stage (Parker HMRB08) that shuttles the sample via a carbon fiber rod (see video in Ref. ^34^). The entire sample (field) trajectory can be programmed, allowing implementation of the polarization, relaxation, and detection periods as in Fig. 1e. Transfer times at the maximum travel range were measured to be 648$\pm$4 ms (see Supplementary Fig. 2), short in comparison with the ${T}_{1n}$ lifetimes we probe. High positional resolution (50 µm) allows access to field steps at high precision (Supplementary Fig. 3 shows full field-position map). The field is primarily in the $\hat{{\bf{z}}}$ direction (parallel to the detection magnet), since sample transport occurs centrally, and the diameter of the shuttling rod (8 mm) is small in comparison with the magnet bore (54 mm). This ensures that the spatial gradients of the magnetic field, both longitudinally, as well as transverse, are negligible (field variation under 1% of the bias field) (see Supplementary Note 2).

### Relaxation rates

In this paper, we perform ${T}_{1}$ noise spectroscopy on ${}^{13}{\rm{C}}$ nuclei in a variety of diamond samples outlined in Table 1. Figure 2 shows representative results, considering here a 10% enriched single crystal (Sample 6). This intriguing data can be visualized in several complementary ways. First, considering relaxation rate ${R}_{1}=1/{T}_{1}$ (Fig. 2b), the high-resolution data allow us to clearly discern three regimes: a steep narrow ${R}_{1}$ increase at ultralow fields (where ultralow refers to the regime approaching zero field $<$10 mT), a broader component at moderate fields (10 mT–500 mT), and an approximately constant relaxation rate independent of field beyond 0.5 T and extending up to 7 T (data beyond 2 T not shown). Each point in Fig. 2b reports the monoexponential decay constant obtained from the full decay curve at every field value (for example shown in Fig. 2c). Error bars at each field value are estimated from monoexponential fits of the polarization decays. The resulting errors are under a few percent. The solid line in Fig. 2b indicates a numerical fit and remarkably closely follows the experimental data. Here, we employ a sum of two Tsallian functions^42,43^ that capture the decay rates at low and moderate fields, and a constant offset at high field (see Fig. 2b insets).

**Table 1:** Summary of samples and defining characteristics. We consider single-crystal (S.C.) samples both at natural abundance and with ${}^{13}{\rm{C}}$ enrichment, as well as diamond particulate (P) samples.

Sample #	[P1] (ppm)	[NV] (ppm)	[${}^{13}{\rm{C}}$] (%)	Particulars	Growth
1	17 $\pm$ 2	$1.4\pm 0.2$	1.1	S.C.	HPHT
2	48 $\pm$ 6	$6.9\pm 0.8$	1.1	S.C.	HPHT
3	$\sim$200	$\sim$1–5	1.1	200 µm P	HPHT
4	$\sim$200	$\sim$1–5	1.1	5 µm P	HPHT
5	$\sim$200	1–10	3	S.C.	CVD
6	$\sim$200	1–10	10	S.C.	CVD
7	$\sim$200	1–10	100	S.C.	CVD

Samples are grown either by high-pressure high-temperature (HPHT) or chemical vapor deposition (CVD) (see Refs. ^29,30^ for sample characterization data)

### Field dependence

A second viewpoint of the data, presented in Fig. 2c, is of the ${T}_{1}$ relaxation times and highlights its highly nonlinear field dependence. There is a step-like behavior in ${T}_{1}({B}_{{\rm{relax}}})$, and an inflection point (knee field) $\approx$100 mT beyond which the ${T}_{1}$’s saturate. We can roughly define the knee-field value, ${B}_{K}^{(1)}$, as the ${B}_{{\rm{relax}}}$ at which the relaxation rate is twice the saturation ${R}_{1}$, although verifying this claim is difficult due to the inherently high relaxometry error at high field. This somewhat counterintutive dependence has significant technological implications. (i) Long ${}^{13}{\rm{C}}$ lifetimes can be fashioned even at relatively modest fields at room temperature. This adds value in the context of ${}^{13}{\rm{C}}$ hyperpolarized nanodiamonds as potential MRI tracers^44^, since it provides enough time for the circulation and binding of surface functionalized particles to illuminate disease conditions. (ii) The step-behavior in Fig. 2c also would prove beneficial for ${}^{13}{\rm{C}}$ hyperpolarization storage and transport. Exceedingly long lifetimes can be obtained by simply translating polarized diamond particles to modest ~100 mT fields—low enough to be produced by simple permanent magnets^40^.

Finally, while the visualizations in Fig. 2b, c cast light on the low and high-field behaviors, respectively, the most natural representation of the wide-field data is on a logarithmic scale (Fig. 2d). The high-density data now unravel the rich relaxation behavior at play in the different field regimes. We discern an additional second inflection point ${B}_{K}^{(2)}$ at lower magnetic fields below which there is a sudden increase in the relaxation rates. The inset in Fig. 2d shows the decomposition into constituent Tsallian fits with a narrow and broad widths.

Microscopic origins of this relaxation behavior can be understood by first considering the diamond lattice to consist of three disjoint spin reservoirs—electron reservoirs of NV centers, P1 centers, and the $${}^{13}{\rm{C}}$$ nuclear spin reservoir. P1 centers arise predominantly during NV center production on account of finite conversion efficiency in the diamond lattice. Indeed the P1 centers are typically at 10–100 times higher concentration than NV centers; with typical lattice concentrations of NVs, P1s, and $${}^{13}{\rm{C}}$$ nuclei, respectively, $${P}_{{\rm{NV}}} \sim$$ 1 ppm, $${P}_{e} \sim$$ 10–100 ppm, and $${P}_{C} \sim 1{0}^{4}\eta$$ ppm, where $$\eta$$ is the $${}^{13}{\rm{C}}$$ lattice enrichment level. At any nonzero field of interest, $${B}_{{\rm{relax}}}$$, the electron, and nuclear reservoirs are centered at widely disparate frequencies and do not overlap. There are also negligible effects of level-anticrossings of NVs to $${}^{13}{\rm{C}}$$ spins, since in all our experiments, the N–V axes are misaligned with the magnetic field. We can separate the relaxation processes in different field regimes to be driven, respectively, by (i) couplings of $${}^{13}{\rm{C}}$$ nuclei to pairs (or generally the reservoir) of P1 centers. This leads to the $${B}_{K}^{(1)}$$ feature at moderate fields in Fig. 2d; (ii) $${}^{13}{\rm{C}}$$ spins interacting with individual P1 or NV centers undergoing lattice-driven relaxation ($${T}_{1e}$$ processes); (iii) inter-nuclear couplings within the $${}^{13}{\rm{C}}$$ reservoir that convert Zeeman order to dipolar order. Both of the latter processes contribute to the low field $${B}_{K}^{(2)}$$ features in Fig. 2d; and finally, (iv) a high-field process $$> $$1 T that shows a slowly varying (approximately constant) field profile. We ascribe this to arise directly or indirectly (via electrons) from two-phonon Raman processes. Since these individual mechanisms are independent, the overall relaxation rate is obtained through a sum, $$\frac{1}{{T}_{1}}={\sum }_{(J)}\frac{1}{{T}_{1}^{(J)}}$$ (shown in the inset of Fig. 2d).

### Effect of electronic spin bath

**Fig. 3:**
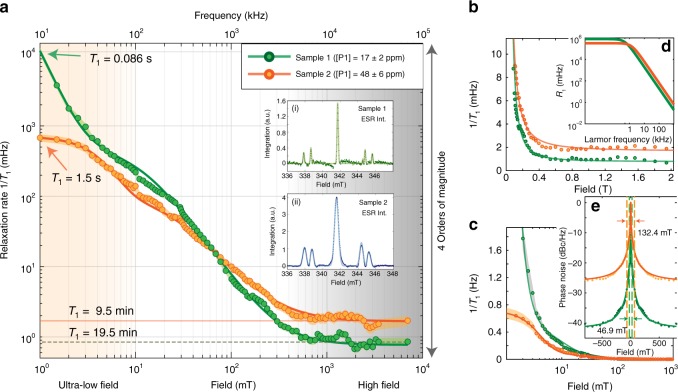
Hyperpolarized relaxometry at natural abundance ${}^{13}{\rm{C}}$ relaxation rate over four decades of field 1 mT-7 T (lower axis) for Samples 1 and 2, probing spin-flipping spectral density from 10 kHz to 75 MHz (upper axis). **a** Relaxation rate on a logarithmic scale, showing steep field dependence that spans four orders of magnitude in ${T}_{1}$, falling to sub-second lifetimes at ultralow fields below ${B}_{K}^{(2)}$, and saturating to lifetimes >10 min. beyond ${B}_{K}^{(1)}$. Orange and green data correspond to CVD samples with different concentration of P1 centers^29^ (legend). Solid lines are fits to a combination of two Tsallian functions. Shaded regions represent error bounds originating from our accelerated data collection strategy (see Supplementary Note 3C). Insets: X-band ESR spectra. **b** High field behavior shows saturating knee field ${B}_{K}^{(1)}$ occurs at higher field for Sample 2. **c** Low field behavior, where intriguingly Sample 2 with a higher P1 concentration has a lower relaxation rate. **d** Calculated relaxation rate ${R}_{1}({\omega }_{L})$ arising from the coupling of the ${}^{13}{\rm{C}}$ spins with the interacting P1 reservoir for the case of 17 ppm (green) and 48 ppm (orange) electron concentrations, showing qualitative agreement with the experimental data from Samples 1 and 2. **e** Comparing effective phase noise ${S}_{p}(\omega )$ for the two samples on a semi-log scale. For clarity, data are mirrored on the *X*-axis and phase noise normalized against relaxation rates at ${\omega }_{0}$ = 1 mT. Solid lines are fits to Tsallian functions. Dashed vertical lines indicate the theoretical widths obtained from the the respective estimates of 2$\left\langle {d}_{ee}\right\rangle$, 46.7 mT, and 131.89 mT, matching very closely the experiments.

Let us first experimentally consider the relaxation process stemming from ${}^{13}{\rm{C}}$ spins coupling to the interacting P1 reservoir. While other lattice paramagnetic defects, e.g., V^−^, N3, and H3 centers^45,46^ are also likely to be present, their concentration is much lower than those of the P1 centers, and their effects to ${}^{13}{\rm{C}}$ relaxation are indistinguishable from the P1s. In Fig. 3, we consider single-crystal samples of natural ${}^{13}{\rm{C}}$ abundance grown under similar conditions but with different nitrogen concentrations (Samples 1–2 in Table 1). Their P1 electron concentrations are ${P}_{e}=$ 17 ppm and ${P}_{e}=$ 48 ppm, respectively, and were measured from X-band ESR^47^ shown in Fig. 3a (inset). To obtain the data with high density of field points, hyperpolarized relaxometry measurements are taken by an accelerated data collection strategy (outlined in Supplementary Note 3) over a ultra-wide-field range from 1 mT-7 T, with DNP being excited at ${B}_{{\rm{pol}}}$ = 36 mT. For relaxometry at fields below ${B}_{{\rm{pol}}}$, we employ rapid current switching of Helmholtz coils within the hyperpolarizer device. Both the range of fields, as well as the density of field points being probed are significantly higher than previous studies^37,48^. This aids in quantitatively unraveling the underlying physics of the relaxation processes. We note that probing relaxation behavior below ~1 mT in our experiments is currently limited by the finite sample shuttling time, which becomes of the order of the ${T}_{1}$’s being probed.

Experimental results in Fig. 3 reveal a remarkably sharp ${R}_{1}$ dependence, best displayed in Fig. 3a on a logarithmic scale, showing variation in relaxation rate over four orders of magnitude. Each curve fits to a sum of two Tsallian functions (solid line), and reveals the ${B}_{K}^{(1)}$ inflection point (closely resembling Fig. 2b) beyond which the lifetimes saturate. The second knee field ${B}_{K}^{(2)}$ at ultralow fields can also be discerned, although determining its exact position is difficult without relaxation data approaching truly zero field. Comparing the two samples (Fig. 3a), we observe a clear correlation in the ${B}_{K}^{(1)}$ knee-field values shifting to higher fields at higher electron concentration ${P}_{e}$. The high-field relaxation rates, highlighted in Fig. 3b, increase with ${P}_{e}$. Interestingly at low-fields (see Fig. 3c), the diamond with lower ${P}_{e}$ (Sample 1) has an enhanced relaxation rate, yielding an apparent “cross-over” in the relaxation data between the two samples at $\approx$50 mT.

**Fig. 4:**
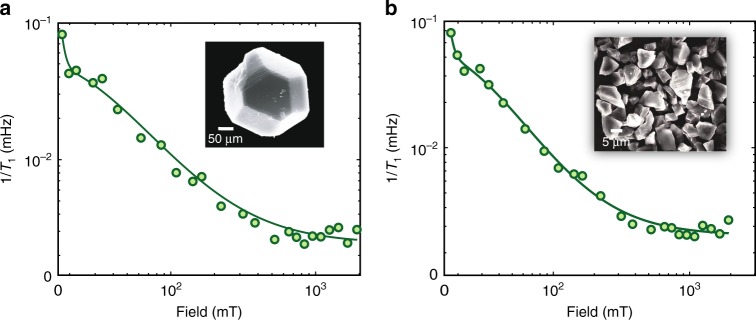
${}^{13}{\rm{C}}$ nuclear relaxation in microdiamond powder. Relaxation-field maps for the randomly oriented natural abundance ${}^{13}{\rm{C}}$ microdiamond powder Samples 3 and 4 of sizes (**a**) 200 µm and (**b**) 5 µm, respectively, with accompanying SEM images (insets). Data are obtained by measuring the full relaxation curve at every field point, and is quantitatively similar to the single-crystal results in Fig. 3.

While we have focused here on single crystals, we observe quantitatively identical relaxation behavior also for microdiamond powders (e.g., Samples 3–4 in Table 1), down to 5-µm sizes (see Fig. 4). Indeed, hyperpolarized particulate samples have a potentially wider application space, both as polarization agents and contrast agents in MRI, and the results indicate that the random orientations of the crystallites play no significant role in the dominant P1-driven nuclear relaxation process. We do expect, however, that for nanodiamond particles $<$100 nm, surface electronic spins will cause an additional relaxation channel.

Let us now develop a simple model to quantify this P1-dominated relaxation process. Given the low relative density of the NV centers and consequently weak NV–NV couplings, to a good approximation they play no role except to inject polarization into the ${}^{13}{\rm{C}}$ nuclei. Consider the Hamiltonian of the system, assumed for simplicity to be a single ${}^{13}{\rm{C}}$ spin, and the environment-interacting bath of P1 centers surrounding it, ${\mathcal{H}}={{\mathcal{H}}}_{S}+{{\mathcal{H}}}_{E}+{{\mathcal{H}}}_{SE}+{{\mathcal{H}}}_{EE}$ where, the first two terms capture the Zeeman parts, the third term is the coupling between reservoirs, and the last term captures the inter-electron dipolar couplings within the P1 bath. Specifically,

1 $${\mathcal{H}}=	\; {\omega }_{L}{I}_{z}+{\omega }_{e}{S}_{z}+\sum_{j}{A}_{zx}^{j}{S}_{zj}{I}_{x}\\ 	+ \sum _{j\,<\, k}{d}_{jk}^{ee}\left[{S}_{zj}{S}_{zk}+\frac{1}{2}\left({S}_{j}^{+}{S}_{k}^{-}+{S}_{j}^{-}{S}_{k}^{+}\right)\right].$$

where $I$ (and $S$) refer to spin-$\frac{1}{2}$ Pauli operators on the nuclei (electrons), respectively, and ${A}_{zx}^{j}$ the pseudo-secular hyperfine interaction that can drive nuclear spin-flips on the ${}^{13}{\rm{C}}$ nuclei. For simplicity, we neglect here the effect of the P1 hyperfine couplings to host ${}^{14}{\rm{N}}$ nuclei. In principle, they just split the electronic reservoirs seen by the ${}^{13}{\rm{C}}$ nuclei into three manifolds separated by the large hyperfine coupling ${A}_{\parallel }^{{\rm{P1}}}\approx$ 114 MHz. In the rotating frame at ${{\mathcal{H}}}_{E}$, and going into an interaction picture with respect to ${{\mathcal{H}}}_{EE}$, the Hamiltonian becomes, ${{\mathcal{H}}}_{I}={\omega }_{L}{I}_{z}+\left\langle {A}_{zx}\right\rangle {I}_{x}{\sum }_{j}\left({e}^{-i{{\mathcal{H}}}_{EE}t}{\hat{S}}_{z}{e}^{i{{\mathcal{H}}}_{EE}t}\right)={\omega }_{L}{I}_{z}+\left\langle {A}_{zx}\right\rangle {\hat{S}}_{z}(t){I}_{x}$ with, $\left\langle {A}_{zx}\right\rangle =\sqrt{\langle {A}_{zx}^{2}\rangle }={[{\sum }_{j}{({A}_{zx}^{j})}^{2}]}^{1/2}$ and the operator ${\hat{S}}_{z}=\frac{1}{\left\langle {A}_{zx}\right\rangle }{\sum }_{j}{A}_{zx}^{j}{S}_{zj}$. Here, $\left\langle {A}_{zx}\right\rangle$ is the total effective P1-${}^{13}{\rm{C}}$ hyperfine interaction, and the norm $\parallel {{\mathcal{H}}}_{EE}\parallel$ is set by the average dipolar interaction between electronic spins in the bath, henceforth $\left\langle {d}_{ee}\right\rangle$. We now make a semi-classical approximation, promoting ${\hat{S}}_{z}$ to ${s}_{z}(t)$, a variable that represents a classical stochastic process seen by the ${}^{13}{\rm{C}}$ nuclear spins^10,49^,

2 $${{\mathcal{H}}}_{I}={\omega }_{L}{I}_{z}+\left\langle {A}_{zx}\right\rangle {s}_{z}(t){I}_{x}\ .$$

In summary therefore, a spin-flipping term ${I}_{x}$ is tethered to a stochastic variable $s(t)$ and this serves as noise on the ${}^{13}{\rm{C}}$ spins, flipping them at random instances, resulting in nuclear relaxation upon a time (or ensemble) average. Interestingly, this noise process arises due to electronic flip-flops in the remote P1 reservoir that are widely separated in frequency from ${}^{13}{\rm{C}}$ spins. In a simplistic picture, shown in Fig. 1c, relaxation originates from pairs of P1 centers in the same ${}^{14}{\rm{N}}$ nuclear manifold (energy-mismatched by $\delta$) undergoing spin flip-flop processes, and flipping a ${}^{13}{\rm{C}}$ nuclear spin (when ${\omega }_{L}\approx \delta$) in order to make up the energy difference. In reality, the overall relaxation is constituted out of the several such processes over the entire P1 electronic spectrum.

Let us now assume the stochastic process ${s}_{z}(t)$ is Gaussian with zero mean and an autocorrelation function $g(\tau )=\exp (-\tau /{\tau }_{c})$ with correlation time ${\tau }_{c}=1/\left\langle {d}_{ee}\right\rangle$. The spectral density function $S(\omega )=\frac{1}{\sqrt{2\pi }}{\int }_{\infty }^{\infty }g(\tau ){e}^{-i\omega \tau }{\mathrm{d}}\tau$ that quantifies the power of the spin-flipping noise components at various frequencies is then a Lorentzian, $S(\omega )=2{\tau }_{c}/(1+{\omega }^{2}{\tau }_{c}^{2})$. Going further now into an interaction picture respect to ${\omega }_{L}{I}_{z}$, ${{\mathcal{H}}}_{I}^{(I)}=\left\langle {A}_{zx}\right\rangle {s}_{z}(t)\left({e}^{-i{\omega }_{L}{I}_{z}t^{\prime} }{I}_{x}{e}^{i{\omega }_{L}{I}_{z}t^{\prime} }\right).$ The survival probability of the spin is, $p(t)=\frac{1}{2}{\rm{Tr}}\{{I}_{z}{e}^{i{{\mathcal{H}}}_{I}^{(I)}t}{I}_{z}{e}^{-i{{\mathcal{H}}}_{I}^{(I)}t}\} \sim {e}^{-\chi (t)}$ where in an average Hamiltonian approximation, retaining effectively time-independent terms, the effective relaxation rate $\chi (t)\approx {R}_{1}t$ just can be obtained by sampling of the spectral density resonant with the nuclear Larmor frequency ${\omega }_{L}$ at each field point. This is the basis behind noise spectroscopy of the underlying ${T}_{1}$ process^50^. We recover then the familiar Bloembergen–Purcell–Pound (BPP) result^51,52^, where the relaxation rate,

3 $$\frac{1}{{T}_{1}^{(1)}}={R}_{1}^{(1)}({\omega }_{L})=\left\langle {A}_{zx}^{2}\right\rangle S({\omega }_{L})=\left\langle {A}_{zx}^{2}\right\rangle \frac{\left\langle {d}_{ee}\right\rangle }{{\omega }_{L}^{2}+{\left\langle {d}_{ee}\right\rangle }^{2}}.$$

**Fig. 5:**
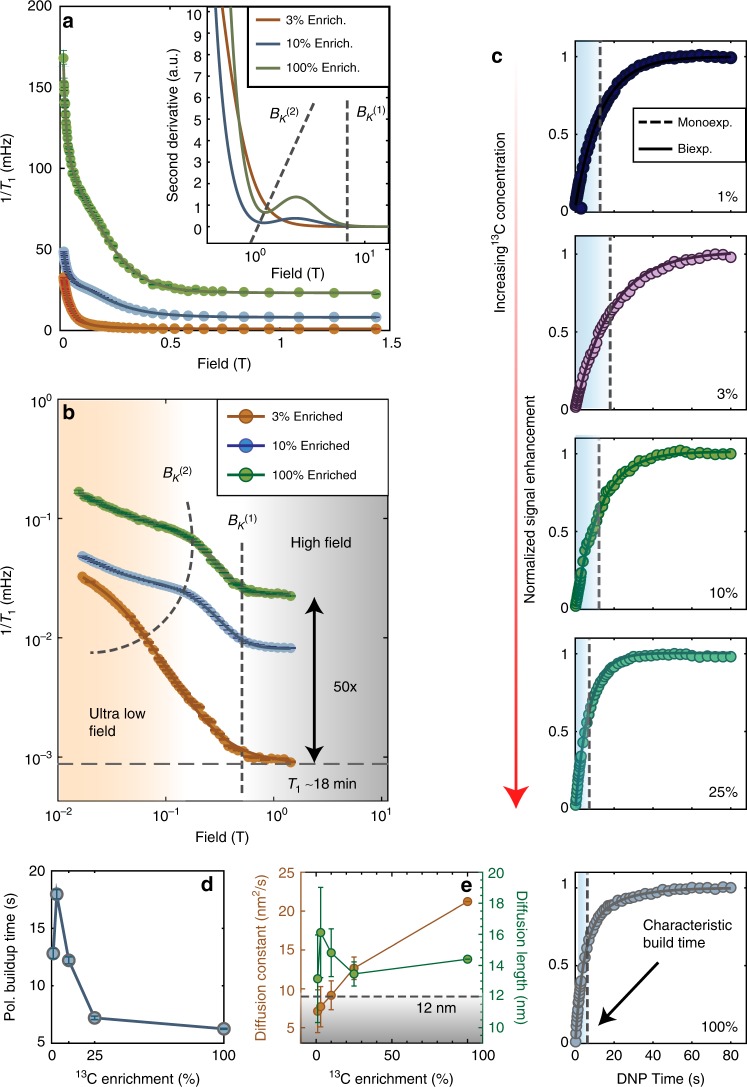
Variation with ${}^{13}{\rm{C}}$ enrichment. Experiments are performed on single crystal samples placed so that all the NV center orientations are identical at 54.7${}^{\circ }$ to ${B}_{{\rm{pol}}}$ = 36 mT. **a** Relaxation rates on linear and (**b**) logarithmic field scale for Samples 5 through 7 with ${}^{13}{\rm{C}}$ concentrations of $3 \%$, $10 \%$, and $100 \%$, respectively, making evident an increase in relaxation rate with increasing ${}^{13}{\rm{C}}$ enrichment at low and high fields. Solid lines are Tsallian fits for each sample. Error bars obtained from confidence intervals of exponential decay curves for the $10 \%$ and $100 \%$ samples, whereas for the $3 \%$ sample the error bars emerge from the accelerated data strategy outlined in Supplementary Fig. 4. Characteristic knee-field ${B}_{K}^{(1)}$ (dashed vertical line) at moderate fields is independent of enrichment, evident in the inset. Knee field at ultralow fields ${B}_{K}^{(2)}$ is qualitatively indicated by the dashed line that serves as a guide to the eye. Inset: Second derivative of the fitted lines, showing the knee fields at the zero-crossings. **c** DNP polarization buildup curves also reflect differences in the nuclear spin lifetimes, displaying saturation at much shorter times upon increasing enrichment. DNP in all curves are performed at 36 mT sweeping the entire ${m}_{s}=+1$ manifold. **d** Polarization buildup times extracted from the data showing that faster nuclear spin relaxation limits the final obtained hyperpolarization enhancements in highly enriched samples. **e** Spin diffusion constant and diffusion length for ${}^{13}{\rm{C}}$ nuclei numerically estimated from the data as a function of lattice enrichment. Dashed line indicates the mean inter-electron distance $\left\langle {r}_{{\rm{NV}}}\right\rangle \approx$ 12 nm between NV centers at 1 ppm concentration, indicating that spin diffusion can homogeneously spread polarization in the lattice almost independent of ${}^{13}{\rm{C}}$ enrichment.

The inter-spin couplings can be estimated from the typical inter-spin distance $\left\langle {r}_{e}\right\rangle ={\left(3/4\pi\, {\mathrm{ln}}\, 2\right)}^{1/3}{N}_{e}^{-1/3}$, where ${N}_{e}=(4\times 1{0}^{-6}{P}_{e})/{a}^{3}$[m^−3^] is the electronic concentration in inverse volume units and $a$ = 0.35 nm the lattice constant of diamond^37^. The couplings are now related to the second moment of the electronic spectra^49^ ${M}_{2e}=\frac{9}{20}{(g{\mu }_{B})}^{2}\frac{1}{{\left\langle {r}_{e}\right\rangle }^{6}},$ where $g\approx 2$ is the electron g-factor, and ${\mu }_{B}=9.27\times 1{0}^{-21}$ erg G^−1^ the Bohr magneton, written in cgs units for convenience in calculation. This gives the Lorentzian estimate $\left\langle {d}_{ee}\right\rangle \approx {\gamma }_{e}\sqrt{\frac{8}{\pi }}\sqrt{{M}_{2e}}$ [Hz] $\approx$ 10.5${P}_{e}$ [mG], that scales approximately linearly with electron concentration ${P}_{e}$^37^. For Samples 1 and 2 with varied ${P}_{e}$, we obtain spectral widths $\left\langle {d}_{ee}\right\rangle$ = 0.5 kHz and 1.42 kHz, respectively, corresponding to field-profile widths of 46.9 mT and 132.4 mT, respectively. These would correspond to inflection points ${B}_{K}^{(1)}=\frac{\left\langle {d}_{ee}\right\rangle }{2{\gamma }_{n}}$ in the relaxometry data at fields 23.5 mT and 66.2 mT, respectively. These values are in remarkable and quantitative agreement with the experimental data (see also Fig. 3e). Moreover, we expect that these turning points (scaling $\propto {P}_{e}$) are independent of ${}^{13}{\rm{C}}$ enrichment $\eta$, in agreement with the data in Fig. 2 (see also Fig. 5).

From lattice considerations (see Supplementary Note 4), we can also estimate the value of the effective hyperfine coupling $\left\langle {A}_{zx}\right\rangle$ in Eq. (3), which we expect to grow slowly with ${P}_{e}$. We make the assumption that there is barrier of ${r}_{0}\approx$ 2.15 nm around every P1 center in which the ${}^{13}{\rm{C}}$ spins are unobservable because their hyperfine shifts exceed the measured ${}^{13}{\rm{C}}$ linewidth $\Delta {f}_{\det }\approx$ 2 kHz. Our estimate can be accomplished by sitting on a P1 spin, and evaluating $\left\langle {A}_{zx}\right\rangle ={\left[\left\langle {A}_{zx}^{2}\right\rangle \right]}^{1/2}$, where the second moment^49^, $\left\langle {A}_{zx}^{2}\right\rangle =\frac{1}{N}{\left[\frac{{\mu }_{0}}{4\pi }{\gamma }_{e}{\gamma }_{n}\hslash \right]}^{2}{\sum }_{j}\frac{{(3\sin {\theta }_{j}\cos {\theta }_{j})}^{2}}{{r}_{j}^{6}}$ with $N$ being the relative number of ${}^{13}{\rm{C}}$ spins per P1 spin, and ${\theta }_{j}$ the angle between the P1-${}^{13}{\rm{C}}$ axis and the magnetic field, and index $j$ runs over the region between neighboring P1 spins. This gives,

4 $$\left\langle {A}_{zx}^{2}\right\rangle \approx {\left(\frac{{\mu }_{0}}{4\pi }{\gamma }_{e}{\gamma }_{n}\hslash \right)}^{2}\frac{6}{5}\frac{1}{{\left\langle {r}_{e}\right\rangle }^{3}}\left(\frac{1}{{r}_{0}^{3}}-\frac{1}{{\left\langle {r}_{e}\right\rangle }^{3}}\right).$$

For the two samples, we have $\left\langle {r}_{e}\right\rangle =$ 4.8 nm and 3.39 nm, respectively, giving rise to the effective P1-${}^{13}{\rm{C}}$ hyperfine interaction $\left\langle {A}_{zx}^{2}\right\rangle \approx$ 0.39 [(kHz)^2^] and $\left\langle {A}_{zx}^{2}\right\rangle \approx$ 0.45 [(kHz)^2^], respectively. These values are also consistent with direct numerical estimates from simulated diamond lattices (see Supplementary Note 4). The simple model stemming from Eq. (2) and Eq. (3) therefore predicts that the effective hyperfine coupling $\left\langle {A}_{zx}\right\rangle$ increases slowly with the electron concentration ${P}_{e}$, with the electron spectral density width $\left\langle {d}_{ee}\right\rangle \propto {P}_{e}$.

Finally, from Eq. (3), we can estimate the zero-field rate stemming from this relaxation process, ${R}_{1}(0)=\frac{\left\langle {A}_{zx}^{2}\right\rangle }{\left\langle {d}_{ee}\right\rangle }\approx$ 777 [s^−1^] and 317.5 [s^−1^], respectively. Figure 3d calculates the resulting relaxation rates from this process ${R}_{1}({\omega }_{L})$ in a logarithmic plot. It shows good semi-quantitative agreement with the data in Fig. 3a and captures the experimental observation that the rates of the two samples cross over at a particular field. It is instructive to represent the data in terms of effective phase noise (see Fig. 3e), denoted logarithmically as, ${S}_{p}({\omega }_{L})=10\mathrm{log}\left(\frac{{R}_{1}({\omega }_{0})}{{R}_{1}({\omega }_{L})}\right)$ [dBc Hz^−1^], where ${\omega }_{0}\to 0$ represents the relaxation rates approaching zero field. Figure 3e shows this for the two samples, employing ${\omega }_{0}=$1 mT, and with the estimated field-linewidths displayed by the dashed lines. This makes evident that the high field spin-flipping noise seen by the ${}^{13}{\rm{C}}$ nuclei is about 15 dB lower in Sample 1.

While Eq. (3) is the dominant relaxation mechanism operational at moderate fields, let us now turn our attention to the the behavior at ultralow fields in Fig. 3. Equation (2) provides the framework to consider the effect of single P1 and NV electrons to the relaxation of ${}^{13}{\rm{C}}$ nuclei. In this case, the stochastic process ${s}_{z}(t)$ arises not on account of inter-electron couplings, but due to individual ${T}_{1e}$ processes operational on the electrons, for instance coupling to lattice phonons. The width of the spectral density is then given by ${T}_{1e}$,

5 $$\frac{1}{{T}_{1}^{(2)}}={R}_{1}^{(2)}({\omega }_{L})=\left\langle {A}_{zx}^{2}\right\rangle \frac{{T}_{1e}}{1+{\omega }_{L}^{2}{T}_{1e}^{2}}.$$

While ${T}_{1e}$ is also field-dependent, and dominated by two-phonon Raman processes at moderate-to-high field, typical values of ${T}_{1e} \sim$ 1 ms^53^, give rise to Lorentzian relaxometry widths of $\approx$1 kHz, corresponding to field turning points of ${B}_{K}^{(2)}\approx \frac{1}{2{\gamma }_{n}{T}_{1e}}=$ 0.1 mT.

Let us finally comment on the role NV centers themselves as a source of ${}^{13}{\rm{C}}$ relaxation. Since NV concentrations are low for samples under study (see Table 1), with NV–NV coupling strengths $\lesssim$25 kHz, relaxation processes stemming from pairs of NV centers have a negligible contribution in comparison to single NV-driven processes. Moreover, close-shell ${}^{13}{\rm{C}}$ nuclei that are most sensitive to this relaxation channel are unobservable in our experiments since they are hyperfine shifted by $\delta \approx \frac{{A}_{zx}\Delta \sin \theta }{{\gamma }_{e}B+\Delta \cos \theta }$ beyond the NMR detection band width $\approx$20 kHz, where $\theta$ is the angle of the N–V axis to the $B$ = 7 T detection field.

### Effect of ${}^{13}{\rm{C}}$ enrichment

To systematically probe this low-field behavior as well as consider the effect of couplings within the ${}^{13}{\rm{C}}$ reservoir, we consider in Fig. 5 diamond crystals with varying ${}^{13}{\rm{C}}$ enrichment $\eta$ and approximately identical NV and P1 concentrations (Samples 5–7). With increasing enrichment, a third relaxation mechanism becomes operational, wherein at low fields it becomes possible to dissipate Zeeman energy into the dipolar bath. The field dependence of this process is expected to be more Gaussian, centered at zero field and have a width $\sim \left\langle {d}_{{\rm{CC}}}\right\rangle$ the mean inter-spin dipolar coupling between ${}^{13}{\rm{C}}$ nuclei. We can estimate these couplings from the second moment, $\left\langle {d}_{{\rm{CC}}}\right\rangle =\frac{1}{N}{\sum }_{j}{[{\sum }_{k}{(\frac{{\mu }_{0}}{4\pi }\hslash {\gamma }_{n}^{2}(3{\cos }^{2}{\theta }_{jk}-1))}^{2}/{r}_{jk}^{6}]}^{1/2}$ where in a lattice of size $\ell$, $N={N}_{C}{\ell }^{3}$ refers to the number of ${}^{13}{\rm{C}}$ spins, and the spin density ${N}_{C}=0.92\eta$ spins nm^−3^. Here, ${\theta }_{jk}={\cos }^{-1}\left(\frac{{{\bf{r}}}_{jk}\cdot {{\bf{B}}}_{{\rm{relax}}}}{{r}_{jk}{B}_{{\rm{relax}}}}\right)$ is the angle between the inter-nuclear vector and the direction of the magnetic field. In the numerical simulations outlined in Supplementary Note 4B, we evaluate the case consistent with experiments wherein the single-crystal samples placed flat, i.e., with ${{\bf{B}}}_{{\rm{relax}}}\parallel$ [001] crystal axis. As a result, for ${}^{13}{\rm{C}}$ spins on adjacent (nearest-neighbor) lattice sites, ${\theta }_{jk}=$ 54.7° is the magic angle and ${d}_{jk}^{{\rm{CC}}}=0$.

We find $\left\langle {d}_{{\rm{CC}}}\right\rangle \approx$ 850 Hz for natural abundance samples and a scaling $\left\langle {d}_{{\rm{CC}}}\right\rangle \propto {\eta }^{1/2}$ with increasing enrichment. This is in good agreement with the experimentally determined linewidths (see Supplementary Note 4). We thus expect a turning point at low fields, ${B}_{K}^{(2)} \sim \frac{\left\langle {d}_{{\rm{CC}}}\right\rangle }{2{\gamma }_{n}}$, for instance $\approx$39 µT for natural abundance samples, but scaling to $\approx$0.46 mT in case of the 100% enriched Sample 7. In real experiments, it is difficult to distinguish between this process and that arising directly from single electrons in Eq. (5), and hence we assign the same label to this field turning point.

Performing hyperpolarized relaxometry (see Fig. 5), we observe that increasing enrichment leads to a fall in nuclear ${T}_{1}$s, evident both at low (Fig. 5a) and high (Fig. 5b) fields. ${R}_{1}$ rates for the diamonds with ${}^{13}{\rm{C}}$ concentrations of $10 \%$ and $100 \%$ (Samples 6 and 7) are obtained by taking the full relaxation decay curves at every field point, while for the $3 \%$ enriched diamond (Sample 5) we use an accelerated data collection strategy (see Supplementary Note 3B) on account of the inherently long ${T}_{1}$ lifetimes. On a logarithmic scale (Fig. 5b), we observe the knee field ${B}_{K}^{(1)}$ is virtually identical across all the samples, indicating it is a feature independent of ${}^{13}{\rm{C}}$ enrichment, originating from interactions with the electronic spin bath. This is in good agreement with the model in Eq. (3). A useful means to evaluate the inflection points arises from the zeros of the second derivative of the Tsallian fits, as indicated in the inset of Fig. 5a. Moreover, the lower inflection field ${B}_{K}^{(2)}$ scales to higher fields with increasing enrichment $\eta$, pointing to its origin from internuclear dipolar effects. At the low fields, we also notice that the samples with lower enrichment have higher relaxation rates, and with steeper field-profile slopes (Fig. 5b). This is once again consistent with the model that the spectral density height and width being probed scales with $\left\langle {d}_{{\rm{CC}}}\right\rangle$.

Changes in the nuclear lifetimes are also reflected directly in the DNP polarization buildup curves, shown in Fig. 5c. We perform here hyperpolarization of all samples under the same conditions, sweeping the entire ${m}_{s}$ = +1 manifold at ${B}_{{\rm{pol}}}$ = 36 mT, sweeping over the full NV ESR spectrum. We note that polarization buildup is predominantly monoexponential (with time constants shown as dashed lines in Fig. 5c), except for at natural abundance ${}^{13}{\rm{C}}$, where biexponential growth (solid lines) is indicative of nuclear spin diffusion. Data demonstrate that highly enriched samples have progressively smaller polarization buildup times (see Fig. 5d) on account of limited nuclear lifetimes at ${B}_{{\rm{pol}}}$.

Moreover, the experimental data allow us to quantify the homogenization of polarization in the lattice. We assign a spin diffusion coefficient $D=\frac{{\left\langle {r}_{n}\right\rangle }^{2}}{30{T}_{2n}}$ (see Fig. 5e), where the ${T}_{2n}$ are evaluated here by only taking the dipolar contribution to the linewidth, ${T}_{2n}\approx 1/\left\langle {d}_{{\rm{CC}}}\right\rangle$^54^. Given a total time bounded by ${T}_{1}$, we can calculate the rms overall diffusion length^55^ as $\sigma =\sqrt{2D{T}_{1}}$ that is displayed as the blue points in Fig. 5e. Also for reference is plotted the mean NV–NV distance $\approx$12 nm at 1 ppm concentration (dashed region in Fig. 5e), indicating that to a good approximation that the optically pumped polarization reaches to all parts of the diamond lattice between the NV centers.

We comment finally that determining the origins of ${}^{13}{\rm{C}}$ relaxation in enriched samples can have several technological applications. Enrichment provides an immediate means to realize quantum registers and sensing modalities constructed out of hybrid NV-${}^{13}{\rm{C}}$ spin clusters, and as such ascertaining nuclear relaxation profiles is of practical importance for such applications. Low $\eta$ ($\le$3%) naturally engender NV-${}^{13}{\rm{C}}$ pairs that can form quantum registers^56–58^. The nuclear spin can serve as an ancillary quantum memory that, when employed in magnetometry applications, can provide significant boosts in sensing resolution^19,59^. With increasing ${}^{13}{\rm{C}}$ concentrations $\eta\; \gtrsim$ 10% a single NV center can be coupled to several ${}^{13}{\rm{C}}$ nuclei forming natural nodes for a quantum information processor, and where the nuclear spins can be actuated directly by hyperfine couplings to the NV electron^60,61^. Approaching full enrichment levels ($\eta =$ 100%), internuclear couplings become significant, permitting hybridized nuclear spin states and decoherence-protected subspaces^62^ for information storage. In bulk quantum sensing too, for instance applied to diamond-based gyroscopes^18,63^, the high density of ${}^{13}{\rm{C}}$ sensor spins (${\sim} {10}^{22}$ cm^−3^), as much as $> 1{0}^{5}$ times the number of NV centers, can be harnessed to increase sensitivity.

## Discussion

**Fig. 6:**
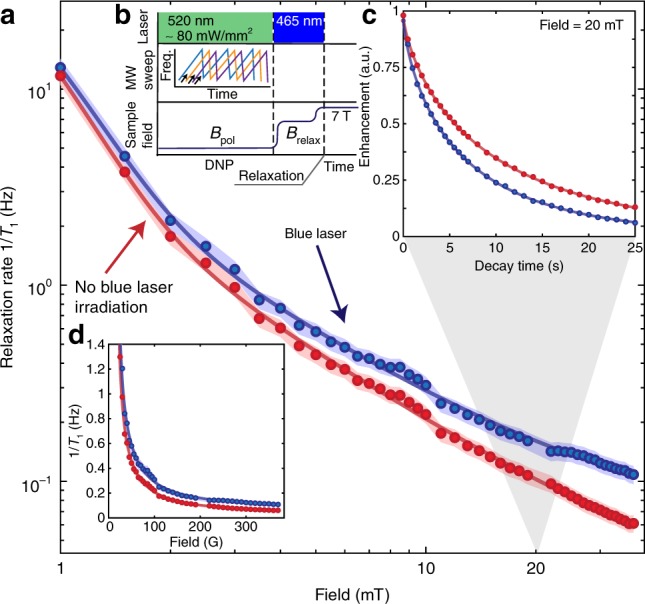
Dynamic optical engineering of electron spin density. Panel **a** denotes ${}^{13}{\rm{C}}$ relaxation rate on a logarithmic scale for Sample 1 employed in Fig. 3 with relaxation dark (red points) and under low power (80 mW mm^−2^) blue (465 nm) irradiation (blue points). Shaded regions represent error bounds (see Supplementary Note 3C). **b** Schematic time sequence of the experiment. **c** Exemplary decay curves obtained at 20 mT. **d** Relaxation rates on linear field scale. We observe that the blue irradiation leads to a decrease in ${}^{13}{\rm{C}}$ nuclear lifetimes, which we hypothesize arises from fluctuations introduced in the electronic spin bath upon recapture after P1 center ionization. This illustrates that the electronic spin spectral density can be optically manipulated, and potentially ultimately also narrowed under sufficiently high-power ionization irradiation.

Experimental results in Fig. 3 and Fig. 5 substantiate the ${}^{13}{\rm{C}}$ relaxation pathways operational at different field regimes, and highlight the important role played by the electronic reservoir toward setting the spin lifetimes. To independently affirm this connection, we perform in Fig. 6 relaxometry under blue ($\lesssim$ 495 nm) wavelengths where the P1 electrons ionize strongly. In these experiments, we operate at the relatively low excitation powers ~240 mW mm^−2^ due to technical limitations related to sample heating. We observe a comparative decrease in nuclear ${T}_{1}$ with respect to decay in the dark (see Fig. 6). In contrast, we do not observe significant change in the lifetimes under 520 nm excitation. We believe that we are accessing an intermediate regime where the P1 ionization rate is slower than the interelectron flip-flop rate by $\left\langle {d}_{ee}\right\rangle$. As a result, ionization is not rapid enough to decouple each spin flip-flop event between neighboring P1 centers. On the contrary, upon ionization and subsequent recapture, the P1 electrons can now cause spin relaxation over a lattice length scale that is longer than purely given by dipole–dipole interactions. This stirring of the electronic spin bath through blue irradiation results in an increased spectral density component at the nuclear Larmor frequency and consequently an increased relaxation rate.

Our experimental observations open the door to a number of intriguing future directions. First, they suggest the prospect of increasing nuclear lifetimes by raising the NV center conversion efficiency^64^. More generally, they point to the efficacy of materials science approaches toward reducing paramagnetic impurities in the lattice. Finally, it opens the possibility of employing coherent quantum control for dissipation engineering, to manipulate the spectral density profile seen by the nuclei and consequently lengthen their ${T}_{1}$. Applying a pulse sequence to increase ${T}_{1}$ has been a longstanding goal in magnetic resonance^65,66^, but is typically intractable because of inability to coherently control broad-spectrum phonon interactions. Instead here since the nuclear ${T}_{1}$ stems from electronic ${T}_{2e}$ processes, these can be echoed out; In particular, the application of electron decoupling (such as WAHUHA^67^ or Lee–Goldburg^68^ decoupling) on the P1 spin bath would suppress the inter-electron flip-flops, narrow the noise spectral density, and consequently shift the knee field ${B}_{K}^{(1)}$ to lower fields. Such ${T}_{1}$ gains just by spin driving at room temperature and without the need for cryogenic cooling, and consequent boosts in the hyperpolarization enhancements—scaling by the decoupling factor—will have far-reaching implications for the optical DNP of liquids under ambient conditions. Given the multi-frequency microwave control driving each of the ${}^{14}{\rm{N}}$ manifolds would entail^69^, an attractive alternate all-optical means is via the optical ionization of P1 centers faster than their flip-flop rate. The exact interplay between optical ionization and recapture rates required for ${T}_{1}$ suppression will be the subject of future work.

Employing hyperpolarized relaxometry, we have mapped the ${}^{13}{\rm{C}}$ nuclear spin lifetimes in a prototypical diamond quantum system over a wide-field range, in natural abundance and enriched ${}^{13}{\rm{C}}$ samples, and for both single crystals as well as powders. We observe a dramatic and intriguing field dependence, where spin lifetimes fall rapidly below a knee field of ~100 mT. The results indicate that the spin lifetimes predominantly arise from nuclear flip processes mediated by the P1 center electronic spin bath, and immediately opens the compelling possibility of boosting nuclear lifetimes by quantum control or optically induced electronic ionization. This has significant implications in quantum sensing, in building longer lived quantum memories, and in practically enhancing the ${}^{13}{\rm{C}}$ hyperpolarization efficiency in diamond, with applications to hyperpolarized imaging of surface functionalized nanodiamonds and for the DNP of liquids brought in contact with high surface area diamond particles.

## Methods

### Materials

Table 1 summarizes the particulars of the samples we employ in this study. ${}^{13}{\rm{C}}$-enriched diamonds (Samples 5–7) used to conduct experiments in Fig. 2 and Fig. 5 were grown through chemical vapor deposition using a ${}^{13}{\rm{C}}$ enrichment mixture of methane and nitrogen (660 ppm, Applied Diamond Inc) as precursor followed by ${}^{13}{\rm{C}}$ enrichments of 10%, 25%, 50%, and 100% to produce the respective percent-enriched diamonds^30^. To produce an NV-concentration of 1–10 ppm, the enriched samples were irradiated with 1 MeV electrons at a fluence of 10^18^ cm^−2^ (Prism Gem LLC) then annealed for 2 h at 800 °C. The natural abundance samples used in Fig. 3 (Samples 1–2) were grown under synthetic high-pressure, high-temperature conditions (Element 6, Sumitomo)^29^ then annealed for 1 hour at 850 °C. The NV and P1 concentration were measured to be 1.4$\pm$0.02 ppm and 17$\pm$2 ppm for the first sample and 6.9$\pm$0.8 ppm and 48$\pm$6 ppm for the second sample, respectively. The microdiamond powders in Fig. 4 (Samples 3–4), produced by HPHT techniques, were acquired from Element 6 and Columbus Nanoworks respectively.

## Supplementary information

Supplementary Information

## Data Availability

The data necessary to replicate the results of this paper are available from the corresponding author upon reasonable request.
