# Supplementary material for: Hyperpolarized relaxometry based nuclear T1 noise spectroscopy in diamond
Source: Nat Commun. 2019 Nov 14;10:5160. doi: 10.1038/s41467-019-13042-3 (PMC6856091; doi:10.1038/s41467-019-13042-3)
Supplement: Supplementary file 1 — Supplementary Information [file 41467_2019_13042_MOESM1_ESM.pdf]

Supplementary Information
Hyperpolarized relaxometry based nuclear T_1 noise spectroscopy in diamond

A. Ajoy,[1] B. Safvati,[1] R. Nazaryan,[1] J. T. Oon,[1] B. Han,[1] P. Raghavan,[1] R. Nirodi,[1] A. Aguilar,[1] K. Liu,[1] X. Cai,[1] X. Lv,[1] E. Druga,[1] C. Ramanathan,[2] J. A. Reimer,[3] C. A. Meriles,[4] D. Suter,[5] and A. Pines[1]

[1] *Department of Chemistry, University of California Berkeley, and Materials Science Division Lawrence Berkeley National Laboratory, Berkeley, California 94720, USA.* [2] *Department of Physics and Astronomy, Dartmouth College, Hanover, New Hampshire 03755, USA.* [3] *Department of Chemical and Biomolecular Engineering, and Materials Science Division Lawrence Berkeley National Laboratory University of California, Berkeley, California 94720, USA.* [4] *Department of Physics, and CUNY-Graduate Center, CUNY-City College of New York, New York, NY 10031, USA.* [5] *Fakultat Physik, Technische Universitat Dortmund, D-44221 Dortmund, Germany.*

SUPPLEMENTARY NOTE 1. EPR MEASUREMENTS IN FIG. 3

EPR spectra of Samples 1 and 2 in Fig.3 were examined with a microwave power 6 mW, averaging over 50 sweeps, with modulation amplitudes of 0.1 mT and 0.01 mT and at sweep fields of 3350 - 3500 G and 3300 - 3600 G for the two samples respectively. Concentrations of P1 centers were estimated by using a $CuSO_4$ reference outlined in Ref. [1].

In order to determine the linewidths of the EPR spectra, a script was written to determine the data range at which Tsallis fits should be applied by first finding the indices where the spectral maxima and minima occured. Midpoints were then determined between the maximum and minimum indices and the first derivative of the Tsallis function was fit to the ranges between the calculated midpoints. Because the baseline was not perfectly zeroed, jumps in the fit values occurred between each range. Applying fits to each individual peak rather than applying one Tsallis function to multiple peaks produced a better baseline correction since the offsets differed between ranges. Each peak was corrected by subtracting the median y-value over the fit range and then making manual corrections if necessary. Once the corrections were completed, the first integrals over each individual range were obtained using trapezoidal integration. The resulting integral arrays were then concatenated and a second integral was obtained. The resulting first integral allowed us to find the line widths of each P1 peak (FWHMs), and the second integral resembled a step function from which the relative step heights of each P1 peak could be found. To account for the hyperfine splittings of the P1 spectra an average over all peaks linewidths was taken and weighted by the height of each peak. The ratio of the averaged linewidths between the two samples in Fig. 3 was found to be 2.97, consistent with the ratio of the P1 concentration of the two samples up to the accuracy of the concentration estimates.

SUPPLEMENTARY NOTE 2. FIELD CYCLING

T_1 noise spectroscopy relies on our ability to rapidly vary the magnetic field experienced by a test sample using a homemade shuttling system built over a 7 T superconducting magnet [2]. Samples are held in an NMR tube (Wilman 8 mm OD, 1 mm thickness) (seeFig. 1D) and pressure-fastened from below the magnet onto a lightweight, carbon fiber shuttling rod (Rock West composites). Using a high precision (50 μm) conveyor actuator stage (Parker HMRB08) (see Fig. 1B), we are able to repeatedly and consistently shuttle from low fields (∼30 mT) below the magnet for polarization to high fields (7 T) within the magnet for NMR detection at sub-second speeds (<700 ms). The instrument is interfaced with a low-cost hyperpolarizer (See [3] for details) , allowing generation and detection of bulk nuclear polarization. Because the average shuttling time is small compared to the nuclear T_1 lifetimes (see Fig. 2) – particularly at fields above 100 mT – our resulting NMR signals are recorded with minimal loss in enhancement. High precision shuttling allowed for the measurement of a full z-direction field map (see Fig. 3)), where the field was measured as a function of position using an axial Hall probe for fields less than 3.5 T. To accommodate the fast shuttling technique, the conventional NMR probe was modified to be hollow, allowing for shuttling through the probe to low magnetic fields below the magnet. Custom made printed coils (see [4]) are employed for direct inductive detection of the NMR signals [2].

From the field map in Fig. 3 it is clear that the gradient experienced by the samples is field-dependent. To quantify the field inhomogeneity during the signal decay process, we numerically estimate the gradient at each height from the acquired field map. The largest gradient occurs when the sample is just entering the bore of the magnet, where the field is around 2 T and the gradient is ∼26 mT mm^{-1}. Because our samples normally occupy a height under 0.5 mm within the sample tube, they will experience a maximum field variation of about 13 mT within the sample. Yet because at this height the magnetic field is so large, this gradient only adds a 0.5% field variation throughout the sample and so this effect can be ignored. For heights closer to the knee fields that we study ∼100 mT, the field gradient is on the order of 1 mT mm^{-1} and so the gradient can be similarly ignored.

SUPPLEMENTARY NOTE 3. DATA PROCESSING

A. Fit models

Nuclear T_1 at a given magnetic field is determined by measuring the decay of NMR signal $\varepsilon(t)$ with respect to time t spent decaying at that field. By measuring the change in signal over various times, relaxation decay curves are determined, and $T_1(B)$ estimated. We find that all the data can be fit to a stretched exponential of the form (see Fig. 4A),

$$\varepsilon(t) = \varepsilon_0 e^{-\left(\frac{t}{T_1(B)}\right)^p}, \tag{1}$$

where $p \in (0, 1]$ is a stretch factor [5], and ϵ_0 represents the bare signal enhancement obtained from DNP and assuming no loss during shuttling. For certain samples, such as the 10% ^{13}C sample in Fig. 2C, we observe that $p \approx 1$, while for most samples with low ^{13}C enrichment (including at natural abundance), $p \in (0.5, 1)$. We ascribe this stretch factor to be arising from spin diffusion of the inhomogeneous polarization in the lattice that is driven by the DNP process.

By measuring the relaxation rate $R_1(B) = 1/T_1(B)$ over a range of magnetic fields allowed by the field cycler, a relaxation field map $R_1(B)$ can be obtained, as shown in Fig. 2B. These relaxation profiles are then fit to a sum of two Tsallis distributions [36], a generalization of Gaussian and Lorentzian functions that

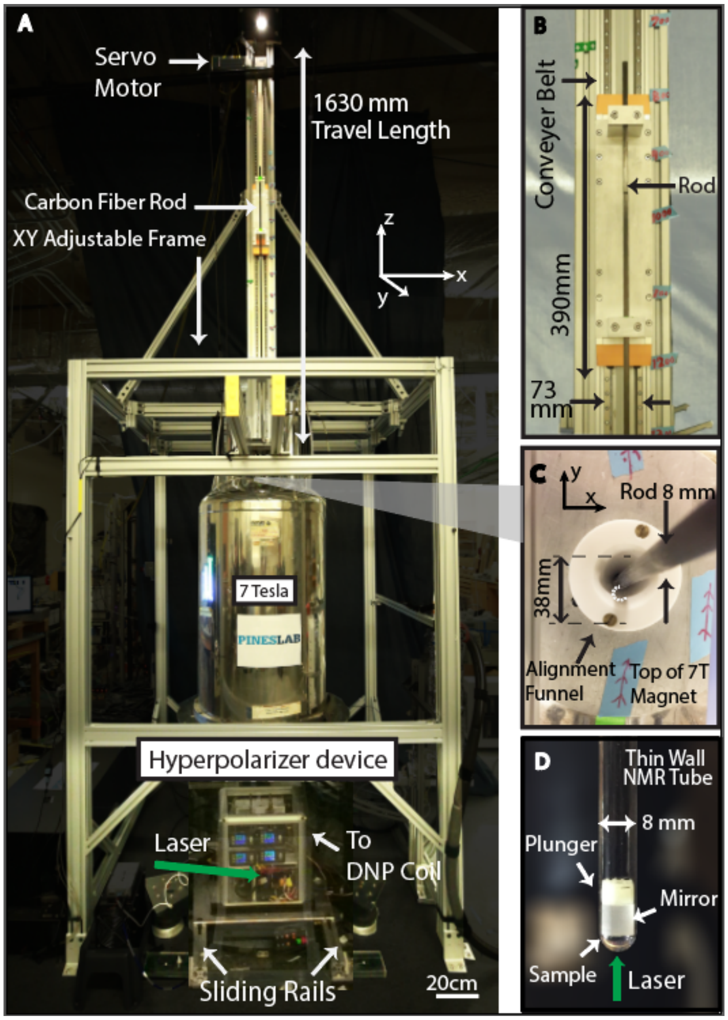

Supplementary Figure 1. **Field cycling device** interfaced with portable diamond hyperpolarizer. (A) Mechanical shuttler is connected to a 7 T magnet and interfaced to a portable hyperpolarizer. Sliding rails attached to the bottom of the device allow for adjustment of hyperpolarizer box and centering of sample above coil. (B) The carbon-fiber shuttling rod is moved along a conveyor belt through use of a twin-carriage actuator. (C) The 8 mm shuttling rod is centered in the 38 mm magnet bore, with a Teflon guide for self-alignment. (D) Diamond sample is held within an 8 mm wide NMR tube, and fitted with a plunger and mirror to prevent excess movement of sample and bolster efficacy of optical pumping.

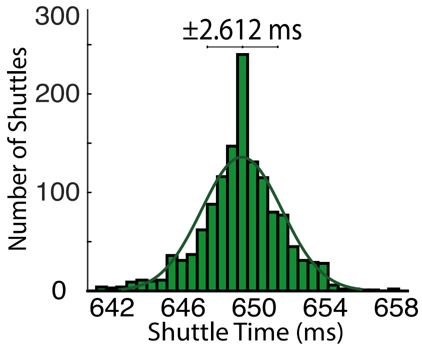

Supplementary Figure 2. **Sample shuttling repeatability.** Shuttler operation (1400 runs) between polarization (∼30 mT) and detection (7 T) locations, distance of approximately 928 mm depending on sample holder inserted. Samples are pressure held onto hollow carbon fiber rod along the center of the magnet bore and shuttled using a mechanical actuator activated by synchronized pulse trigger. This demonstrates high stability for repeated experiments, with average travel times of 648±0.6 ms.

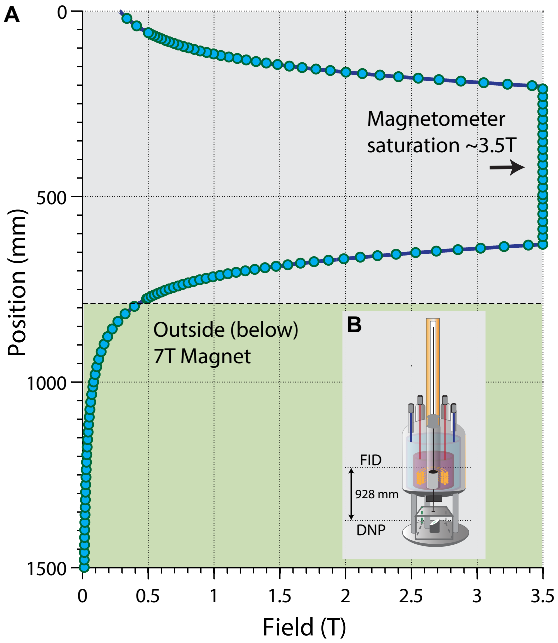

Supplementary Figure 3. **Field map** (A) Measurement of longitudinal (z axis) magnetic field over full field cycler range using a sensitive magnetometer. Data points were attained by shuttling magnetic field probe through center of the magnetic bore while held within the hollow carbon fiber shuttling rod, limiting accuracy to the 50 μm precision of the actuator. Position of magnet entrance is shown to demonstrate fringe field profile. Due to magnetometer constraints, high field measurements saturate at 3.5 T. (B) Polarization is generated ∼928 mm from the NMR coil, depending on the sample holder. This range can be traveled in sub-second speeds (see Fig. 1), allowing fast transport of hyperpolarized diamonds from low fields below to center of magnet with minimal relaxation loss.

allows greater flexibility in representing the relaxation rate as a function of field. Additionally our model assumes a constant offset to account for the saturation of the relaxation rate at high field, with functional form of a single Tsallian with respect to field B,

$$R_1(B) = C_1 \left[1 + (2^{q-1} - 1) \left(\frac{B}{C_2} \right)^2 \right]^{-\frac{1}{q-1}} + C_3 \qquad (2)$$

where fitting parameters C_1, C_2, C_3 describe the amplitude, width and vertical offset of the function respectively, and q regulates the effective contribution of the function's tail to the overall area under the function, with pointwise limits $q = 1$ and $q = 2$ denoting Gaussian and Lorentzian functions respectively. Originally the fitting models were limited to either Lorentzian/Gaussian lineshapes, and the model was susceptible to deviate from the experimental relaxation estimates at high field. By allowing variation of the parameter q, qualitatively better fits to the relaxation profiles can be found and analyzed in relation to one another.

B. Accelerated data collection strategy

We describe here an accelerated technique for data acquisition that we employ in experiments for long-T_1 samples. We work on the principle that one does not necessarily have to obtain the entire relaxation curve (with substantial time costs) in order to accurately measure the relaxation rates. Instead, employing an appropriate (stretched exponential) model, and with the measure-

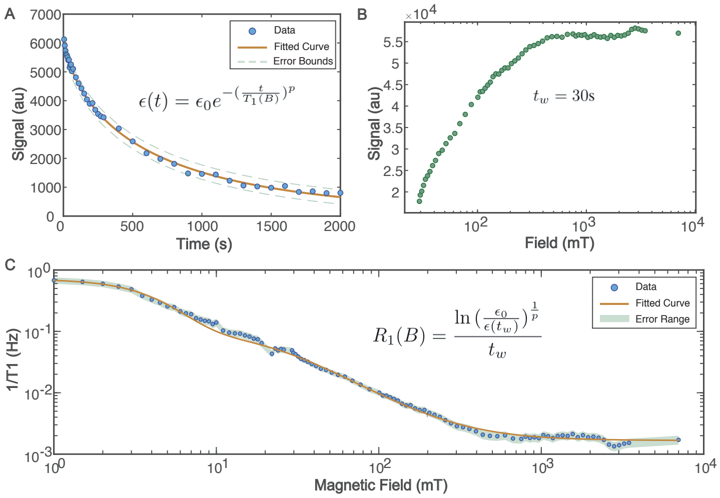

Supplementary Figure 4. **Data processing.** (A) Spin polarization decay curves are acquired by repeated hyperpolarization of the diamond sample followed by time-dependent relaxation at a given field. By varying wait time and measuring the resulting NMR signal, relaxation parameters at this field can be estimated by fitting the data to a stretched exponential function. Because the relaxation rate equation incorporates a phenomenological stretch factor to account for T1 heterogeneity at different fields, decay experiments are done at varied fields and the fitted parameters are used for different field regimes. (B) enhancement data is also taken at varying fields with wait time kept constant, providing a 1D slice of the relaxation dynamics at wide field ranges. To maximize signal contrast the wait times can be dynamically adjusted to account for particularly smaller relaxation times at lower fields. (C) Using the two previous experiments, a relaxation field map is constructed using the estimated rate equation parameters and the 1D enhancement data. Errors result from the quality of the decay curve fits and inaccuracies in the measured magnetic field.

ment of signal losses at fixed selections of waiting times allows the relaxation rates to be reconstructed with low error.

To be more precise, consider that due to long relaxation times at high field, occasionally approaching ∼20 minutes, production of enhancement decay data at an array of magnetic fields is time-intensive. In order to hasten measurement times, and to obtain a denser map of nuclear T_1 estimates at a large number (∼100) of field points (for example in Fig. 3), after hyperpolarization and subsequent transfer to the field of interest, the signal $\varepsilon(t_w)$ after some fixed wait time t_w (typically 30 s) is measured. Fig. 4 details the procedure and benchmarks it. Indeed, the polarization decay is field-dependent, and the set of enhancement values obtained in this manner allows a quantification of the the relaxation mechanisms throughout the full field range. To estimate T_1 from this data, however, requires knowledge of the enhancement generated before relaxation begins. To estimate this quantity, hereafter referred to as ε_0, decay curves are experimentally acquired at certain fields using several averages per experiment, ensuring low error when fitting this curve to a stretched exponential model. Using the fit parameters T_1 and p, ε_0 can then be estimated as

$$\varepsilon_0 = \varepsilon(t_w) e^{\left(\frac{t_w}{T_1}\right)^p} \tag{3}$$

This estimate allows us to reconstruct the relaxation rate at each field for which enhancement measurements were acquired. Importantly, this process removes the need to construct a decay curve like that seen in Fig. 4A for each point of interest. Instead, decay curves for a subset of the field points can be found and the fit parameters from those curves can be used to approximate the relaxation rate for the points in Fig. 4B where an explicit decay curve is not created. By reordering the relaxation equation, the estimate of R_1 at field B becomes

$$R_1(B) = \frac{\ln\left[\frac{\varepsilon_0}{\varepsilon(t_w)}\right]^{\frac{1}{p}}}{t_w} \tag{4}$$

The quality of this reconstruction is improved by generating multiple decay curve experiments at varying fields so that the appropriate initial enhancements ε_0 and stretch factors p can be determined for different field regimes. For the two natural abundance ^{13}C samples in Fig. 3 (Samples 1-2 in Table I) we used decay curve data at fields of 20 mT, 35 mT, 150 mT, and 7 T for the relaxation field map reconstructions, with stretch factors $p \approx 0.75$ at lower fields and $p \approx 1$ at high fields. For the enriched samples in Fig. 5 (Samples 5-7 in Table I), the approximation method was used for the 3% sample whereas the other sample data was acquired using the longer 2D decay curve procedure.

In certain cases, especially for the ultra-low field data in Fig. 3, rather than using a constant decay time t_w for all points, the sensitivity of the decayed enhancement readings is maximized by using dynamically varied wait times t_w at different fields; the loss in enhancement then becomes approximately 50% of the initial polarization value. This process mitigates errors in the measured enhancement values by creating sufficient contrast between the initial and decayed enhancement values, without excessively diminishing the signal relative to the noise.

Let us now quantify the time savings resulting from this data collection strategy. By removing the need to explicitly plot the signal decay over time at every magnetic field point, the effective dimensionality of our $T_1(B)$ measurement process is reduced, which allows determination of T_1 at a large number of field points rapidly. To develop an intuition for the accelerated in the averaging time gained as a result, we assume an even sampling of the signal decay, in time increments Δt across n steps. To obtain estimates of T_1 at N field values, this would require at the very least a total time $t_{2D} = N\Delta t \sum_i^n i = N\Delta t \frac{n(n+1)}{2}$. While employing the accelerated 1D measurement strategy in contrast, signal enhancement is measured after a fixed wait time t_w at each field. These measurements are obtained at all N field points, after sampling with high accuracy the signal decay curves at N_d overlapping fields to construct estimates of the initial enhancement and stretch factor at varied fields. The experiment would therefore expend a minimum time of $t_{1\mathrm{D}} = Nt_{\mathrm{w}} + N_d\Delta t\frac{n(n+1)}{2}$. This measurement strategy incurs a theoretical time gain of $\frac{t_{2\mathrm{D}}}{t_{1\mathrm{D}}} = \frac{N\Delta tn(n+1)}{2Nt_w + N_d\Delta tn(n+1)}$, with the simplifying assumption that zero time is spent moving between fields as well as during signal detection. To demonstrate the possible time gains of this method, assume signal decay measurements at $\Delta t = 10$ s increments for a total of $n = 40$ points in time, across $N = 100$ field points. This may then be compared to the accelerated 1D measurement strategy, with signal enhancement measurements after a fixed hyperpolarization time of t_w = 30 s at each field. If $N_d = 4$ decay curves are used to estimate the relevant relaxation properties at four separate fields, the time gain of the 1D strategy is $\frac{t_{2\mathrm{D}}}{t_{1\mathrm{D}}} \approx 23$.

C. Error estimates

Let us now outline the error estimation in the $T_1(B)$ data. The primary sources of error come from the tightness of the decay curve fits to estimate ε_0 and p at different fields, the shot-to-shot error in the measured enhancement, and the error in the wait time spent relaxing at a given field. Because of the high averaging done to generate relaxation decay curves, the error in ε_0 and p, taken from the fitting function confidence intervals, is very small $\approx$1%. To account for variation in the relaxation wait time, the two methods used for placing the sample at a given field are considered. To access high field points the sample is shuttled into the magnet and allowed to wait a set time, and the error in this process arises from the shuttling time. Because the field cycler can shuttle the sample over the maximum field range in less than 1 second, the shuttling error is approximated as 2 s. To access the low field regime, a bidirectional Helmholtz coil was assembled within the hyperpolarizer which is aligned with the field produced by the superconducting magnet in the $+z$ direction. This allows us to probe fields lower than what is covered by the field cycler. At the polarization location and with no current driven through the coil, the 7 T magnet produces a field of 20.8 mT, but fields as low as 1 mT and even further can be attained with use of the coil. To account for the build-up of magnetic field due to the coil, we attribute an error of 2s to all points found by this process. In combining both shuttled and coil-generated field points there was a constant offset of 15 mT added to all shuttled field points to make the curves consistent with the low field relaxation rate points.

SUPPLEMENTARY NOTE 4. MODEL FOR HYPERPOLARIZED RELAXOMETRY

We now provide more details of the model employed to capture the relaxation mechanisms probed by our experiments. We had identified from the experiments three relaxation channels that are operational at different field regimes, driven respectively by *(i)* couplings of the ^{13}C nuclei to pairs (or generally the reservoir) of P1 center, *(ii)* individual P1 or NV centers, and *(iii)* due to spin-diffusion effects within the ^{13}C reservoir. In this section, we detail lattice calculations that allow the estimation of the spectral densities in each of these cases.

Consider again the three disjoint spin reservoirs in the diamond lattice, the electron spin reservoir of NV centers, electron reservoir of substitutional-nitrogen (P1 centers), and the ^{13}C nuclear spin reservoir. They are centered respectively at frequencies $\omega_{\rm NV} \approx [(\Delta \pm \gamma_e B_{\rm relax}\cos\theta_{\rm NV})^2 + (\gamma_e B_{\rm relax}\sin\theta_{\rm NV})^2]^{1/2}$, $\omega_e \approx [(\gamma_e B_{\rm relax} + m_I A_{\parallel}^{\rm P1}\cos\theta_{\rm P1})^2 + (m_I A_{\perp}^{\rm P1}\sin\theta_{\rm P1})^2]^{1/2}$ and the nuclear Larmor frequency $\omega_L = \gamma_n B_{\rm relax}$; where $\theta_{\rm NV}, \theta_{\rm P1}$ are angles of the NV(P1) axes to the field, $A_{\parallel}^{\rm P1} \approx 114$ MHz, $A_{\perp}^{\rm P1} \approx 86$ MHz are the hyperfine field of the P1 center to its host ^{14}N nuclear spin, $m_I = \{-1, 0, 1\}$ is the ^{14}N manifold, Δ = 2.87 GHz is the NV center zero field splitting, and $\gamma_e = 2.8$ MHz G^{-1} and $\gamma_n = 1.07$ kHz G^{-1} are the electronic and nuclear gyromagnetic ratios.

A. Lattice estimates for electron reservoir

In order to determine the relaxation in behavior Eq. (3) quantitatively, let us determine typical inter-spin couplings and distances for the electron reservoir from lattice concentrations. First, for the electronic spins, given the relatively low concentrations, and the fact that the lattice is populated independently and randomly, we make a Poisson approximation following Ref. [6]. An estimate for the typical inter-spin distance $\langle r_e\rangle$ is obtained by determining the distance at which the probability of finding zero particles is $\frac{1}{2}$. Given the lattice constant of diamond a = 0.35 nm, and the fact that there are four atoms per unit cell, we can estimate the electronic concentration in inverse volume units as, $N_e = (4\times 10^{-6}P_e)/a^3$ [m^{-3}]. Then from the Poisson approximation $\langle r_e\rangle = (3/4\pi\ln 2)^{1/3}\, N_e^{-1/3}$ we obtain, for instance, $\langle r_{\rm NV}\rangle = 12.12$ nm and $\langle r_{\rm P1}\rangle = 2.61$ nm, where we have assumed concentrations of 1 ppm and 100 ppm respectively.

The inter-spin distances now allow us to calculate the second moment of the electronic spectra, which are reflective of the mean inter-spin couplings. Following Abragam [7], we have

$$M_{2e} = \frac{9}{20}(g\mu_B)^2\frac{1}{\langle r_e\rangle^6}, \tag{5}$$

where $g \approx 2$ is the electron g-factor, and $\mu_B = 9.27\times 10^{-21}$ erg G^{-1} the Bohr magneton written in cgs units for convenience. Substituting this leads to, $M_{2e} = 43.65P_e^2$ [mG^2], and allows us to estimate the electronic line width, $\Delta f_e = \langle d_{ee}\rangle \approx \gamma_e\sqrt{\frac{8}{\pi}}\sqrt{M_{2e}}$ [Hz] $\approx 10.5P_e$ [mG], that scales approximately linearly with electron concentration P_e. Here we have assumed a Lorentzian lineshape and quantified the linewidth from the first derivative [6]. Typical values are $\Delta f_{\rm NV}$ = 29.52 kHz and $\Delta f_{\rm P1}$ = 2.95 MHz at 1 ppm and 100 ppm concentrations respectively.

Let us now estimate the effective hyperfine interaction from the P1 centers to the ^{13}C reservoir. Our estimate can be accomplished by sitting on a P1 spin, and evaluating the mean perpendicular hyperfine coupling that contributes to the spin flipping noise, $\langle A_{zx}\rangle = \left[\langle A_{zx}^2\rangle\right]^{1/2}$, where we setup the second moment sum,

$$\langle A_{zx}^2\rangle = \frac{1}{N}\left[\frac{\mu_0}{4\pi}\gamma_e\gamma_n\hbar\right]^2\sum_j\frac{(3\sin\theta_j\cos\theta_j)^2}{r_j^6} \tag{6}$$

where N is the total number of ^{13}C spins for every P1 center and θ_j is the angle between the P1-^{13}C axis and the magnetic field. Numerically the factor $\frac{\mu_0}{4\pi}\gamma_e\gamma_n\hbar = 19.79$ [kHz (nm)^3]. For simplicity, we can approximate the sum by an integral, and including the density of ^{13}C spins $N_C = 0.92\eta$ spins nm^{-3} (see Fig. 6B), where η is the ^{13}C enrichment level,

$$\langle A_{zx}^2\rangle = \left(\frac{\mu_0}{4\pi}\gamma_e\gamma_n\hbar\right)^2\frac{N_C(2\pi)}{N_C\mathcal{V}}\int_{r_0}^{\langle r_e\rangle}\int_0^{\pi/2}\frac{(9\sin^3\theta\cos^2\theta)}{r^6}r^2drd\theta$$

where $\mathcal{V} = \frac{4\pi}{3}\langle r_e\rangle^3$ corresponds to the volume of spins considered. We have assumed that the "sphere of influence" of a particular P1 spin notionally extends to the mean distance between neighboring P1 centers, for instance $\langle r_e\rangle$ = 5.62 nm for P_e = 10 ppm. The integral lower limit is set by the requirement that the hyperfine shift of the ^{13}C nuclei is within the detected NMR linewidth $\Delta f_{\rm det} \approx 2$ kHz. Then, $r_0 = [19.79/(\Delta f_{\rm det})]^{1/3} \approx 2.15$ nm. In principle, r_0 goes to quantify a "barrier" around around each P1 center, wherein the hyperfine interactions prevent the ^{13}C nuclei from being directly observable in our relaxometry experiments. The angle part of the integral evaluates to $6/5$, and effectively therefore,

$$\langle A_{zx}^2\rangle = \left(\frac{\mu_0}{4\pi}\gamma_e\gamma_n\hbar\right)^2\frac{6}{5}\frac{1}{\langle r_e\rangle^3}\left(\frac{1}{r_0^3} - \frac{1}{\langle r_e\rangle^3}\right) \tag{7}$$

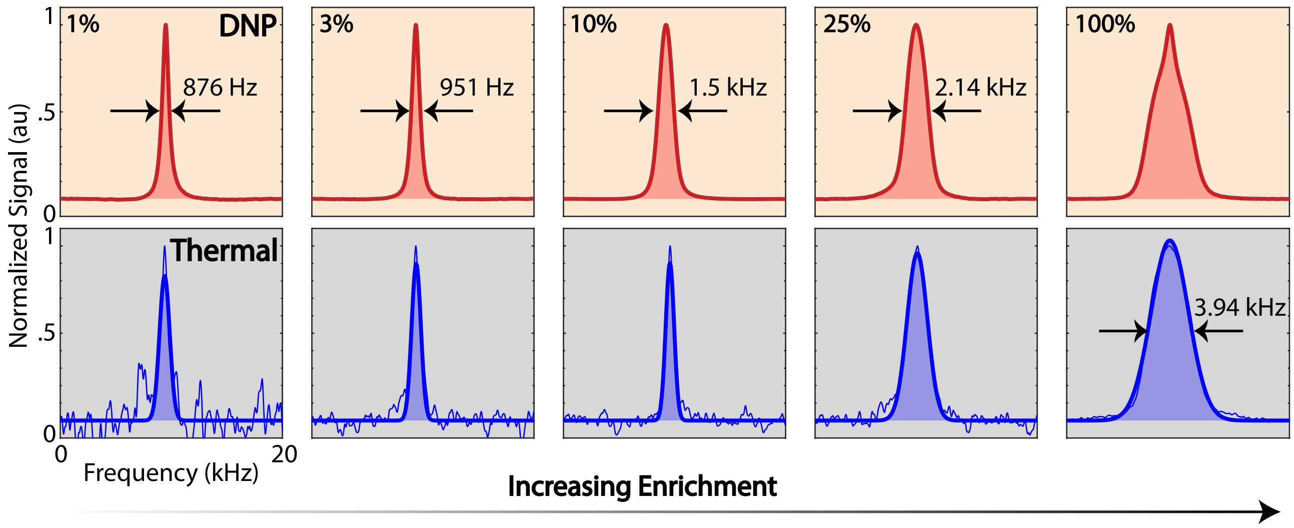

Supplementary Figure 5. **Comparison of DNP and thermal ^{13}C lineshapes.** Panels indicate lineshapes under (A) hyperpolarization carried out at low field (1-30 mT) and (B) 7 T thermal polarization. DNP is excited from the optically polarized NV centers which are ≈1 ppm in all samples. For the 100% sample, we ascribe the broad and narrow components of the lineshapes (dashed lines) as being spins close and further away from the NV centers respectively. The scaling of the experimental linewidths matches our predictions from theory (see Fig. 6C).

For instance, for the two natural abundance single crystal samples that we considered in the Fig. **??** of the main paper with P1 concentration 17 ppm and 48 ppm, we have $\langle r_e \rangle = 4.8$ nm and 3.39 nm respectively, giving rise to the effective P1-^{13}C hyperfine interaction $\langle A_{zx}^2 \rangle \approx 0.39$ [(kHz)^2] and $\langle A_{zx}^2 \rangle \approx 0.45$ [(kHz)^2] respectively. The simple model predicts that the effective hyperfine coupling increases slowly with the electron concentration P_e, that the electron spectral density width $\langle d_{ee} \rangle \propto P_e$. It also shows that the electron spectral density is independent of ^{13}C enrichment η to first order. The zero-field relaxation rates stemming from this coupled-electron mechanism can now be calculated as $R_1(0) = \langle A_{zx}^2 \rangle / \langle d_{ee} \rangle \approx 777$ [s^{-1}] and 317.5 [s^{-1}]. This matches our expectation for the order of magnitude of the zero field rate since we expect that the ^{13}C relaxation time T_{1n} matches that of the electron $T_{1e} \approx 1$ ms.

In order to validate the conclusions from this simple model, we perform an alternative numerical estimation of $\langle A_{zx}^2 \rangle = \left[\frac{1}{N} \sum_{j \in \Delta f_{\text{det}}} \langle A_{zx,j}^2 \rangle \right]$ within the detection barrier directly from the diamond lattice (see Fig. 6F and Supplementary Note 4C). We obtain $\langle A_{zx}^2 \rangle = 2$ [(kHz)^2] and 2.26 [(kHz)^2] for Samples 1 and 2 respectively, in close and quantitative agreement with the values predicted from Eq. (7) (considering the approximations made in the analysis). Numerics also confirm that the hyperfine values $\langle A_{zx}^2 \rangle$ are independent of enrichment η (see Fig. 6F) in agreement with the experimental data.

B. Lattice estimates for ^{13}C reservoir

In contrast, since the ^{13}C reservoir has a much larger spin density, especially at high enrichment levels, we will estimate the interspin distances $\langle r_n \rangle$ and couplings Δf_n numerically. The experimentally obtained ^{13}C lineshapes and resulting linewidths for all the samples considered are shown in Fig. 5. We begin by first setting up a diamond lattice numerically and populating the ^{13}C spins with enrichment level set by η. The numerical calculation is tractable since only small lattice sizes typically under ℓ=10 nm are sufficient to ensure convergence of the various dipolar parameters (see Fig. 6A). To a good approximation, we determine the spin density of the ^{13}C nuclei to be $N_C = 0.92\eta$ spins nm^{-3} (see Fig. 6B). Next, in order to determine the nuclear dipolar linewidths, we consider the secular dipolar interaction between two nuclear spins j and k in lattice,

$$d_{jk}^{\text{CC}} = \frac{\mu_0}{4\pi} \hbar \gamma_n^2 (3\cos^2\theta_{jk} - 1) \frac{1}{r_{jk}^3} \tag{8}$$

where $\theta_{jk} = \cos^{-1}\left(\frac{\mathbf{r}_{jk} \cdot \mathbf{B}_{\text{pol}}}{r_{jk} B_{\text{pol}}} \right)$ is the angle between the internuclear vector and the direction of the magnetic field. In the numerical simulations we will consider, we evaluate the case of single crystal samples placed flat, i.e. with $\mathbf{B}_{\text{pol}} \parallel [001]$ crystal axis. As a result, for ^{13}C spins on adjacent lattice sites, $\theta_{jk} = 54.7°$ is the magic angle and $d_{jk}^{\text{CC}} = 0$. We note that Eq. (8) is a good approximation even during the hyperpolarization process. Indeed, although hyperpolarization is performed in the regime where the nuclear Larmor frequency ω_L is smaller than the hyperfine interaction A to the NV center, the hyperfine field is only transiently on during the microwave sweep. Given the fact that the NV center is a spin-1 electron, there is no hyperfine field applied to the nuclei when the NV is optically pumped to the $m_s = 0$ spin state. Indeed this constitutes the majority of time period of the DNP process.

We now evaluate the effective mean dipolar coupling $\langle d_{\text{CC}} \rangle$ between the nuclei from the second moment,

$$\langle d_{\text{CC}} \rangle = \frac{1}{N} \sum_j \left[\sum_k \left(\frac{\mu_0}{4\pi} \hbar \gamma_n^2 (3\cos^2\theta_{jk} - 1) \right)^2 \frac{1}{r_{jk}^6} \right]^{1/2}, \tag{9}$$

where $N = N_C \ell^3$ refers to the number of ^{13}C spins in the lattice, and for the convergence, we assign for simplicity, $1/r_{jj} = 0$. This simply allows us to sum over all the spins j in the lattice. In practice, we evaluate the parameter $\langle d_{\text{CC}} \rangle$ in Eq. (9) over several (≈ 20) realizations of the lattice and take an ensemble average (see Fig. 6C). We report an effective error bar from the standard deviation of this distribution. The fidelity of the obtained results is evaluated by testing the convergence $\epsilon(\ell) = \| \langle d_{\text{CC}} \rangle^{\ell+1} - \langle d_{\text{CC}} \rangle^{\ell} \|$,

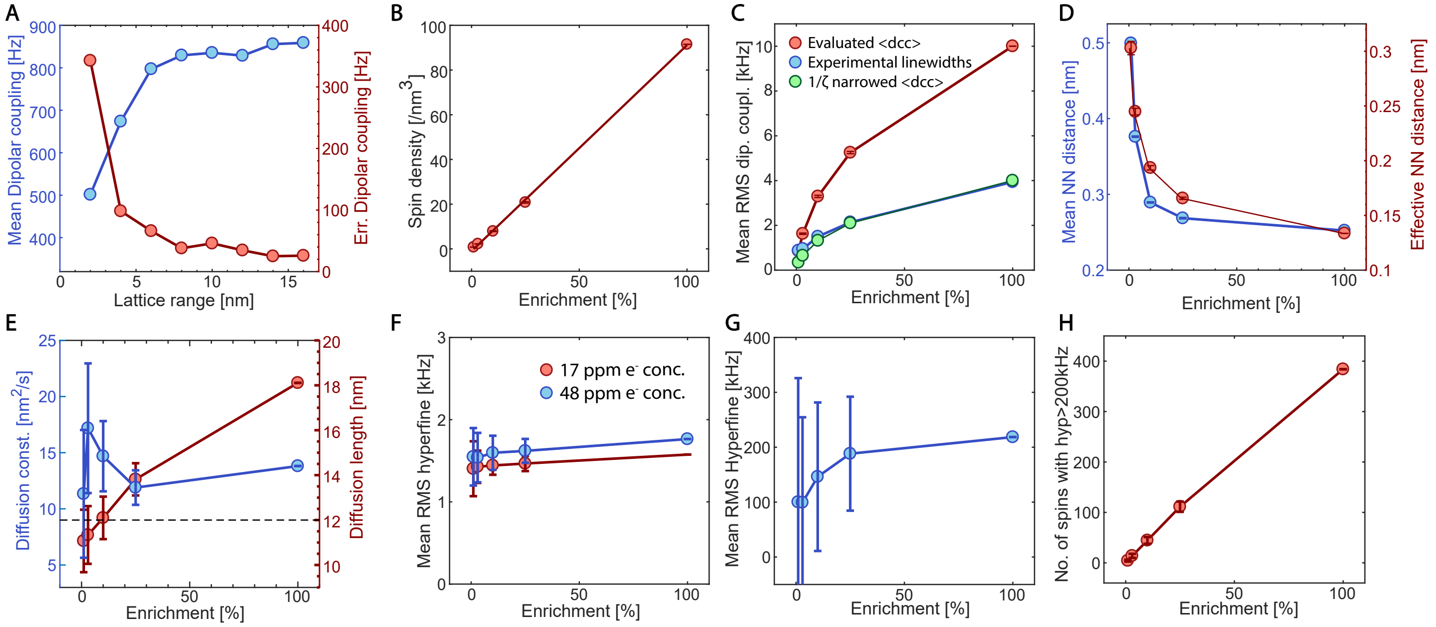

Supplementary Figure 6. **Calculated interspin parameters pertaining to ^{13}C and NV reservoirs as a function of lattice enrichment η.** (A) *Convergence of numerical estimates* is representatively illustrated by plotting the mean ^{13}C dipolar coupling $\langle d_{\rm CC}\rangle^{\ell}$ and the residual $\epsilon(\ell)$ as a function of considered lattice size ℓ. We evaluated here the case of a 1% enriched diamond single crystal. We observe good convergence beyond a lattice size of about 10 nm. (B) *Spin density* of ^{13}C nuclei shows, as expected, very close to linear dependence with η. Solid line is a linear fit, whose slope returns the lattice spin density $\approx 0.92\eta$ spins nm^{-3}. (C) *Effective inter-nuclear dipolar coupling* $\langle d_{\rm CC}\rangle$ evaluated from second moment (red line). Blue points show the experimentally obtained linewidths. Green line indicates $\frac{1}{\zeta}\langle d_{\rm CC}\rangle$ with broadening factor $\zeta = 2.5$, and shows a good numerical agreement with experimental data. (D) *Mean inter-spin distance* $\langle r_n\rangle$ between lattice ^{13}C nuclei is evaluated from the RMS dipolar coupling (red points) and from effective nearest-neighbor lattice distances (blue points). The two estimates show a good match, with the inter-spin distance falling approximately as $\eta^{1/3}$. (E) *Diffusion constant and diffusion length* numerically estimated with lattice enrichment. Here we employed experimentally obtained values of ^{13}C T1. Dashed line indicates the mean inter-electron distance between NV centers at 1 ppm concentration, indicating that spin diffusion can homogeneously spread polarization in the lattice almost independent of ^{13}C enrichment. (F) *Effective hyperfine coupling* $\langle A_{zx}^{\rm obs}\rangle$ to P1 centers in case of single crystal samples with 17 ppm (red points) and 48 ppm (blue points) electron concentration. Results indicate that $\langle A_{zx}^{\rm obs}\rangle$ is independent of ^{13}C enrichment η. (G) *Estimates of mean RMS NV-^{13}C hyperfine interaction* $\langle A_{\rm NV}\rangle$ with lattice enrichment. (H) *Estimation of directly participating ^{13}C nuclei* in the DNP process, defined as those nuclei for which the hyperfine coupling to the closest NV center is greater than 200 kHz. We obtain an approximately linear increase with enrichment. Error bars in all panels are numerically estimated from standard deviation of lattice parameter distributions over several realizations of the lattice configuration.

where the $(\ell+1)$ superscript indicates a lattice expanded by 1nm. As is evident in the representative example for $\eta = 1.1\%$ displayed in Fig. 6A, we find good convergence ($\epsilon \to 0$) for $\ell \approx 14$ nm, corresponding to about 2500 lattice ^{13}C nuclei.

It is instructive to now compare the estimated values with the experimentally determined nuclear linewidths $\Delta f_n(\eta)$ measured at 7 T (see Fig. 5 and blue points in Fig. 6C). The scaling (solid line in Fig. 6C) of the experimental data $\sim \eta^{1/2}$ matches closely with the estimated result through Eq. (9) (see red line in Fig. 6C). However we find that the numerical value overestimates the linewidth by an additional broadening factor $\zeta \approx 2.5$. The green points show a close match between experimental values and numerically evaluated $\frac{1}{\zeta}\langle d_{\rm CC}\rangle$.

This effective coupling now allows us to estimate the mean inter-spin distance $\langle r_n\rangle$ as a function of ^{13}C enrichment (see Fig. 6D),

$$\langle r_n\rangle = \left[\frac{2\langle d_{\rm CC}\rangle}{\frac{\mu_0}{4\pi}\gamma_n^2\hbar}\right]^{-1/3} \quad (10)$$

We find a scaling $\sim \eta^{-1/6}$ (red line in Fig. 6D). It is also interesting to compare these values to those alternatively evaluated directly from the lattice (blue points in Fig. 6C). For this, we rely on the fact that the $\langle r_n\rangle$ distances largely reflect the nearest-neighbor (NN) spin distances. We define the NN spin (say k) to the spin j as the one which has the dipolar coupling d_{jk} is maximal. Now for every spin j in the lattice, we determine the nearest neighbor inter-spin distance $R_j = \left|r_{jk}^{\rm NN}\right|$, and construct a row matrix, $\mathbf{R} = \{R_j\}$, with $j^{\rm th}$ element R_j. Finally, repeating and contacentating this row matrix for several realizations of the lattice, we estimate $\langle r_n\rangle = \langle \mathbf{R}\rangle$ for the i$^{\rm th}$ realization of the lattice. The comparison between these two metrics is demonstrated in Fig. 6D), and show reasonably good agreement.

These inter-spin distances and the coupling values allow us to estimate the spin diffusion coefficient $D(\eta)$ as a function of lattice enrichment (see Fig. 6E). This quantifies the spread of polarization away from directly polarized ^{13}C nuclei, and also serves as a means to quantify the homogenization of polarization in the lattice. Following Ref. [8], we heuristically assign a spin diffusion coefficient $D = \frac{\langle r_n\rangle^2}{30T_{2n}}$ where the T_{2n} are evaluated here by only taking the dipolar contribution to the linewidth, $T_{2n} \approx 1/\langle d_{\rm CC}\rangle$. Given a total time bounded by T_1, we can calculate the rms overall diffusion length [9] as $\sigma = \sqrt{2DT_1}$ that is displayed as the blue points in Fig. 6D. Also for reference is plotted the mean NV-NV distance ≈ 12 nm at 1 ppm concentration, indicating that to a good approximation that the optically pumped polarization reaches to all parts of the diamond lattice between the NV centers.

C. Lattice estimates for hyperfine couplings to NV and P1 reservoirs

Let us finally evaluate, through similar numerical means, details of the hyperfine interaction between ^{13}C reservoir and the electron reservoirs of the P1 centers and NV centers. We draw a distinction between the NV and P1 centers in the fact that the former are spin-1, with a nonmagnetic $m_s = 0$ state (with no hyperfine coupling to first order), while the latter are spin $1/2$. When hyperfine shifts exceed the observed 7 T NMR linewidth Δf_{det} ∼2 kHz, it is safe to assume that these spins are unobservable - a case that is operational more strongly for the spin $1/2$ P1 centers.

In order to perform the estimation, in the generated lattice of size $\ell = \langle r_e \rangle$, we populate ^{13}C spins with enrichment η, and include an electron at the lattice origin. The mean perpendicular hyperfine interaction between P1-^{13}C spins is calculated from the second moment, from the individual hyperfine couplings $A_{zx,j}$ that are smaller than the detection barrier Δf_{det}

$$\langle A_{zx}^{\text{obs}} \rangle = \left[\sum_{j \in \text{obs}} \langle A_{zx,j}^2 \rangle \right]^{1/2} = \left[\frac{1}{N_{\text{obs}}} \sum_{j \in \text{obs}} \left(\frac{\mu_0}{4\pi} \gamma_e \gamma_n \hbar \right)^2 \frac{(3 \sin\theta_j \cos\theta_j)^2}{r_j^6} \right]^{1/2} \tag{11}$$

where N_{obs} refers to the number of spins amongst the total $N = N_C \ell^3$ spins for which $\langle A_{zx,j}^2 \rangle < (\Delta f_{\text{det}})^2$. Here r_j is the distance of the jth ^{13}C nucleus, and θ_j the angle of P1-^{13}C axis to the magnetic field, and we have ignored the effect of ^{14}N hyperfine interactions intrinsic to the P1 center. This effective hyperfine field, scaling with lattice enrichment η, is then indicated by the red (blue) points in Fig. 6F for electron concentrations of 17 ppm (48 ppm) respectively. The error bars display the standard deviation of the obtained distributions upon several hundred realizations of the lattice. We observe that the effective hyperfine interaction $\langle A_{zx}^{\text{obs}} \rangle$ is almost independent of η, and is higher for lattices with higher P_e electron concentration. This is consistent with the results obtained through Eq. (7) and matches our experimental observations in Fig. 5 of the main paper. For natural abundance samples we numerically obtain $\langle A_{zx}^{\text{obs}} \rangle$ = 1.4 kHz, 1.55 kHz, and 1.04 kHz respectively for 17 ppm, 48 ppm, and 1 ppm (representative of NV center concentrations), in agreement with estimates from Eq. (7).

Finally, let us estimate the number of spins that are directly polarized by the NV centers. In Fig. 6G we evaluate the *full* hyperfine interaction to ^{13}C spins of varying enrichment, considering no operational detection barrier.

$$\langle A_{\text{NV}} \rangle = -\left[\sum_j \langle A_{j,\text{NV}}^2 \rangle \right]^{1/2} = \left[\frac{1}{N} \sum_j \left(\frac{\mu_0}{4\pi} \gamma_e \gamma_n \hbar \right)^2 \cdot \frac{[(3r_{jz}^2 - 1)^2 + (3r_{jx}r_{jz})^2 + (3r_{jy}r_{jz})^2]}{r_j^6} \right]^{1/2}$$

where we employed a lattice size $\ell = \langle r_{\text{NV}} \rangle$ = 12 nm, and $N = N_C \ell^3$ refers to the number of ^{13}C spins in the lattice with index j running over all them. Here the angle part of the hyperfine interaction is evaluated by assigning the direction cosines, for instance as, $r_{jz} = (\vec{r_j} \cdot \hat{z}_{\text{NV}})/r_j$, where $\hat{z}_{\text{NV}}$ is the unit vector aligned along the N-V axis, collinear with the direction of the strong zero field splitting that forms the dominant part of the Hamiltonian at low fields. This effective hyperfine field, scaling with lattice enrichment η, is then indicated by the blue points in Fig. 6G. Our DNP mechanism is a low-field one and is primarily effective when the full hyperfine coupling $\langle A_{j,\text{NV}} \rangle$ is of the order of greater than the nuclear Larmor frequency $\omega_L = \gamma_n B_{\text{pol}}$, where B_{pol} is the polarizing field. We can heuristically measure the number of directly polarized spins surrounding an NV center as being those for which $\langle A_{j,\text{NV}} \rangle > 200$ kHz. As Fig. 6H indicates, the number of such directly polarized nuclei scales approximately linearly with ^{13}C enrichment, with a constant ratio $\approx 4.3\eta$ in the diamond lattice. Spin diffusion therefore plays an important role in the spread of polarization away from these directly polarized nuclei.

[1] E. Scott, M. Drake, and J. A. Reimer, Journal of Magnetic Resonance **264**, 154 (2016).

[2] A. Ajoy, X. Lv, E. Druga, K. Liu, B. Safvati, A. Morabe, M. Fenton, R. Nazaryan, S. Patel, T. F. Sjolander, J. A. Reimer, D. Sakellariou, C. A. Meriles, and A. Pines, Review of Scientific Instruments **90**, 013112 (2019), https://doi.org/10.1063/1.5064685.

[3] A. Ajoy, R. Nazaryan, E. Druga, K. Liu, A. Aguilar, B. Han, M. Gierth, J. T. Oon, B. Safvati, R. Tsang, *et al.*, arXiv preprint arXiv:1811.10218 (2018).

[4] Video showing method of "printing" coils for inductive spin readout, https://www.youtube.com/watch?v=7oP7KERSoNM/.

[5] A. Jarmola, V. Acosta, K. Jensen, S. Chemerisov, and D. Budker, Physical review letters **108**, 197601 (2012).

[6] E. Reynhardt, Concepts in Magnetic Resonance Part A **19A**, 20 (2003).

[7] A. Abragam, *Principles of Nuclear Magnetism* (Oxford Univ. Press, 1961).

[8] H. Hayashi, K. M. Itoh, and L. S. Vlasenko, Physical Review B **78**, 153201 (2008).

[9] W. Zhang and D. G. Cory, Phys. Rev. Lett. **80**, 1324 (1998).
